# Comparative Numerical Evaluation of Feed-Spacer Geometries in Reverse Osmosis Modules for Enhanced Water Treatment Sustainability

**DOI:** 10.3390/membranes16080265

**Published:** 2026-08-10

**Authors:** Hussain Al-Sairfi, Fajer M. Alelaj, Mohammad K. Alhamli, Mustafa Fadel, Hawraa Sabti

**Affiliations:** 1 Water Research Center, Kuwait Institute for Scientific Research (KISR), Safat Square, P.O. Box 24885, Kuwait City 13109, Kuwait; felaj@kisr.edu.kw; 2 Department of Mechanical Power and Refrigeration Tech (MPR), College of Technological Studies (CTS), Public Authority for Applied Education and Training (PAAET), Kuwait City 70030, Kuwait; mk.alhamli@paaet.edu.kw; 3 Electrical Engineering Department, College of Engineering, Mustansiriyah University, Baghdad P.O. Box 10001, Iraq; mustafa_1988abbas@uomustansiriyah.edu.iq; 4 Environmental Affairs Department, Kuwait Municipality, Safat Square, P.O. Box 10, Kuwait City 13001, Kuwait; eng.h.sabti@hotmail.com

**Keywords:** Computational Fluid Dynamics (CFD), reverse osmosis, concentration polarization, sustainable water treatment, Digital Twin, additive manufacturing

## Abstract

The lack of freshwater in the world requires a paradigm shift from linear water consumption to resilient and low-energy desalination technologies. Although reverse osmosis (RO) is the standard in the industry, its usefulness is essentially constrained by concentration polarization (CP) and non-useful hydraulic pressure losses. This paper applies a high-fidelity computational model in ANSYS Fluent 2022 R1 to conduct a comparative parametric evaluation of hexagonal and sinusoidal feed-spacer geometries relative to a baseline grid configuration. The solute concentration gradients at the fluid–membrane interface were solved using a 3D species transport model, which was optimized using one-micron near-wall inflation layers. The hexagonal configuration produced the lowest maximum membrane-surface salt mass fraction, decreasing it from 0.1127 kg/kg for the baseline grid to 0.0429 kg/kg, corresponding to a 61.9% reduction. Although the hexagonal design required an inlet pressure of 205.7 Pa, it produced a more favorable normalized mass-transfer–friction trade-off than the sinusoidal configuration (447.8 Pa), with a System Performance Index (η) of 2.53. These results demonstrate comparative micro-scale improvements in concentration polarization control and hydraulic performance under the simulated conditions. Experimental testing and system-level modeling are required before conclusions can be drawn regarding full-module energy consumption, photovoltaic integration, long-term fouling behavior, or economic feasibility. This study is consistent with the emerging Concepts and design for sustainability, whereby a circular and energy-efficient water economy is facilitated through an innovative mechanical design.

## 1. Introduction

The world today is at a critical crossroads, where industrialization and freshwater security directly oppose each other. Clean water and sanitation are core needs for stability in the world, as expressed in the United Nations Sustainable Development Goal 6 (SDG 6). Nevertheless, since the number of people in the world who have no access to water is about 1.1 billion, and there are 2.7 billion people who can count on having no water for a month per year, the use of non-conventional water sources has ceased to be an optional strategy and has now become the default one [1,2,3,4].

The most popular industrial standard for curbing this crisis has been the use of desalination technology, especially reverse osmosis (RO). The current RO systems supply more than 65 percent of the desalinated water in the world because of their scalability and lower energy use as compared to thermal desalination processes. Although these benefits exist, the physics of the fluid–membrane interface limits the efficiency of RO operations. The fact that solute builds up at the membrane surface, coupled with the amount of hydraulic energy loss required to permeate flux, is a major obstacle on the way to a truly circular water economy [1,2,3,4,5].

The operational efficiency of RO is not an isolated mechanical variable, as it is deeply integrated with its energy source. In the Arabian Peninsula, environmental stressors, such as air pollution, have been shown to cause significant economic losses in grid-connected PV systems [6,7]. Maximizing the performance ratio of these solar arrays through tilt-angle optimization and irradiation analysis is essential to ensure a consistent power supply for desalination [8,9,10]. Spacer designs with lower hydraulic resistance may be relevant to desalination systems operating under variable energy availability; however, this relationship requires full-system energy modeling beyond the channel-scale CFD analysis used in this study.

High-fidelity numerical modeling allows mechanical components to be evaluated before physical prototyping. The present research applies this approach to examine micro-scale flows and mass-transfer processes in RO channels, focusing on a comparative assessment of the selected feed-spacer geometries [11].

### 1.1. Problem Statement

The main technical limitation of reverse osmosis operation is the so-called concentration polarization (CP). When water is pressed through a semi-permeable membrane under high pressure, the salt ions are rejected and form a dense layer of solution near the membrane surface. This regional rise in salinity increases the osmotic pressure that must be overcome, thus increasing the feed pressures required to hold a constant permeate flux [12,13,14,15].

The conventional industrial practice for reducing CP is the incorporation of feed spacers in the flow channels. These mesh-like structures disturb the developing boundary layer and generate localized flow separation, recirculation, reattachment, and secondary motion, thereby enhancing mixing near the membrane surface. The introduction of such spacers, however, introduces a trade-off of parasitism: [16,17,18]

- Hydraulic Resistance: Spacers block the flow passage, causing large pressure drops across the module.
- Dead Zones: Stagnant zones due to poorly optimized geometries occur between filament contact points, serving as breeding grounds for biofouling and mineral scaling [19,20,21,22,23,24].
- Specific Energy Consumption (SEC): The additional energy needed to pump against resistance due to the spacers directly adds to the carbon footprint of the water treatment facility.

Current grid-based designs of spacers do not tend to reconcile these opposing requirements, either providing too little mixing or too much hydraulic drag. The spacer geometry, particularly hexagonal or bio-inspired sinusoidal structures, urgently requires “Emerging Concepts” capable of attaining even better mass transfer while reducing energy costs even further [25,26,27,28,29].

The provision of clean water is no longer a localized issue but a requirement for global stability. Therefore, improving RO channel-scale hydrodynamics remains important for reducing concentration polarization and avoiding unnecessary hydraulic losses under the simulated conditions.

### 1.2. Study Aim

The proposed research will fill the gap between highly developed mechanical designs and sustainable water treatments by conducting a comprehensive numerical study. Using a Digital Twin approach in ANSYS Fluent, the study aims to achieve the following specific objectives:

- Create a High-Fidelity Numerical Workflow: To create a 3D species transport model capable of resolving the one-micron boundary layer with a 10-layer inflation mesh, such that the concentration gradient is resolved with peer-reviewed accuracy.
- Measure Geometric Performance Indicators: To perform a comparative study of traditional grid spacers with more advanced hexagonal and sinusoidal geometries; the Concentration Polarization Modulus (Γ) and Pressure Increase Factor (fp) are some of the key performance indicators that will need to be measured.
- Identify Energy-Efficient Blueprints: To identify which geometries provide a good balance between mitigation and energy use, and to provide a data-driven blueprint of the future generation of 3D-printed sustainable feed spacers.

This research will help the ECDS mission of ensuring a digital shift in mechanical engineering to enable a sustainable and energy-saving global system by achieving these goals.

## 2. Literature Review

Since the early commercialization of reverse osmosis, feed spacers in spiral-wound membrane (SWM) modules have been used. Traditionally, spacers were simple plastic diamond mesh extrusions. The initial studies by [14,16,17,18,24,25,26,27,28,29] determined that, although these structures were needed to ensure that the membrane leaves were not in contact with each other, they were the main reason for losses in hydraulic efficiency. The early 2000s’ literature paid much attention to the dead zones that were generated in the filament crossover points. These areas, with zero or near-zero velocity, were found to be the most common causes for biofouling and mineral scaling. This emphasis shifted as the industry shifted toward sustainable water treatment, and simple separation was replaced by active mixing; where the spacer is considered, a mechanical part is created to maximize mass transfer using the least amount of energy.

The most important technical challenge of RO technology is still concentration polarization. The film theory, as derived in early membrane science, states that a layer of repelled solutes form on the membrane surface, developing an osmotic pressure gradient that counteracts the applied feed pressure [12,13,14,15]. The Concentration Polarization Modulus (Γ) is the main indicator of process efficiency, and has previously been determined in the literature [12,13,14,15]. Recent investigations have pointed out that Γ is not a fixed value but a local quantity that varies according to spacer geometry. Studies of the Sherwood number (Sh), a non-dimensional ratio of convective-to-diffusive mass transport, have found that non-conventional geometries can enhance Sh by up to 40 percent of that of empty channels, although this is at the expense of a higher friction factor (f).

Advances in computational resources have enabled the transition from simplified two-dimensional models to three-dimensional CFD simulations of flow and mass transfer in spacer-filled membrane channels. The appropriate flow formulation depends on the Reynolds-number range and spacer configuration. Turbulence closures, including k-ω SST, may be applicable under a sufficiently high Reynolds-number range or transitional conditions. However, the present study examined Reynolds numbers of approximately 70–350 and therefore used the laminar model. The vortical structures generated around the spacer filaments are interpreted as geometry-induced laminar secondary flows caused by separation and reattachment rather than as turbulence predicted through a turbulence-closure model [30,31,32,33,34,35,36].

The literature has experienced an upsurge in Emerging Concepts that are not based on a grid in the past decade. Additional geometries explored in this study are bio-inspired geometries, which aim to emulate flow patterns in nature to minimize skin friction. Equally, hexagonal architectures have been suggested as a way of enhancing the scouring area of membranes without a lot of augmentation of the volume fraction of spacers. Recent work in additive manufacturing has shown that these high-performance and complex geometries are now able to be produced at high-precision levels. This study expands on the literature through direct quantification of the ratio of mitigation to energy use of these advanced shapes, addressing a gap in the literature on the energy footprint of hexagonal spacers in high-salinity seawater applications [37,38,39,40,41,42,43].

The chemical environment of brine plays a decisive role in membrane longevity. Modeling with concentrated brine solutions suggests that the ionic strength significantly influences mineral solubility limits [44]. It is essential that the design of spacers will not lead to localized precipitation and enable the extraction of some minerals from a mixed brine solution, such that the waste can be used as a resource [45]. Previous studies have also examined spacer orientation and geometry as a means of generating secondary flow and improving near-wall mass transfer in the membrane channels [25,26,27,28,29,30,31,32,33,34,35,36,37,38,39,40,41,42,43]. The present study contributes to this body of work by comparing the mass-transfer and hydraulic resistance characteristics of grid, hexagonal, and sinusoidal configurations under identical high-salinity operating conditions.

## 3. Methodology

The numerical study was carried out in a three-dimensional fluid space that was the unit cell of a reverse osmosis (RO) module. To ensure consistency across all parametric studies, a uniform computational domain was used with a length of 15 mm, a width of 9.6 mm, and a height of 0.8 mm.

### 3.1. Mesh Independence and Spatial Convergence

A systematic mesh sensitivity analysis was carried out to make sure that the numerical solutions did not depend on the spatial discretization. This was done to check that the gradients of velocity and concentration, which are to be captured, are due to the physical model and not a grid density artifact. As per normal CFD practice for membrane systems, the mesh was refined, particularly in the high-gradient areas around the walls of the membrane, using a specific inflation strategy. Three different mesh densities, i.e., Coarse, Medium, and Fine, were considered to evaluate at which point the solution had reached asymptotic stability. The main parameters monitored during this study were the maximal mass fraction of NaCl ($C_{max}$) at the membrane interface and the total pressure drop (∆P) across the domain of 15 mm. This convergence study has been summarized in Table 1 to present the quantitative results obtained [30,31,32,33,34,35,36].

The change from the Medium to Fine mesh density did not lead to a significant change of more than 1.22 and 0.80 percent in the predicted salt concentration and hydraulic pressure drop, respectively, as shown in Table 1. The Fine mesh was further evaluated for mesh quality metrics to ensure solver robustness. The average element skewness was 0.21 (well below the recommended threshold of 0.85), confirming that the polyhedral elements within the hexagonal filament vicinity did not introduce significant angular distortion. The maximum aspect ratio was 12.4, which was acceptable for the inflation layers near the membrane wall. The inflation layer growth rate was set to 1.2, producing 10 layers with a first-layer height of 1 × 10^−6^ m (1 μm) to ensure y^+^ < 1 across all membrane surfaces. These quality parameters are consistent with the best-practice guidelines for species transport CFD in membrane channels. In numerical studies, a deviation of less than 2 percent is usually viewed as representative of a mesh-independent solution. As a result, the Fine mesh parameters (around 1.8 million elements) became the norm for all future Digital Twin assessments, such as the hexagonal and sinusoidal ones. Such a choice guarantees a high level of numerical integrity, allowing the identification of small-scale geometric optimizations with the precision required by the sustainability objectives of the ECDS 2026 conference [30,31,32,33,34,35,36].

Mesh independence demonstrates that the numerical outputs are not materially dependent on the selected grid density; it does not, by itself, constitute experimental validation. As an additional benchmark assessment, the baseline grid results were compared with the published friction-factor and Sherwood-number correlations reported by Koutsou et al. under comparable spacer-filled channel conditions. The calculated deviations were approximately 4.3% for the friction factor and 4.7% for the Sherwood number. This agreement supports the reasonableness of the baseline flow and mass-transfer formulation. However, the advanced hexagonal and sinusoidal geometries have not yet been validated experimentally, and their predicted performance should therefore be interpreted as comparative numerical evidence [15].

### 3.2. Geometric Configuration

**Figure 1:**
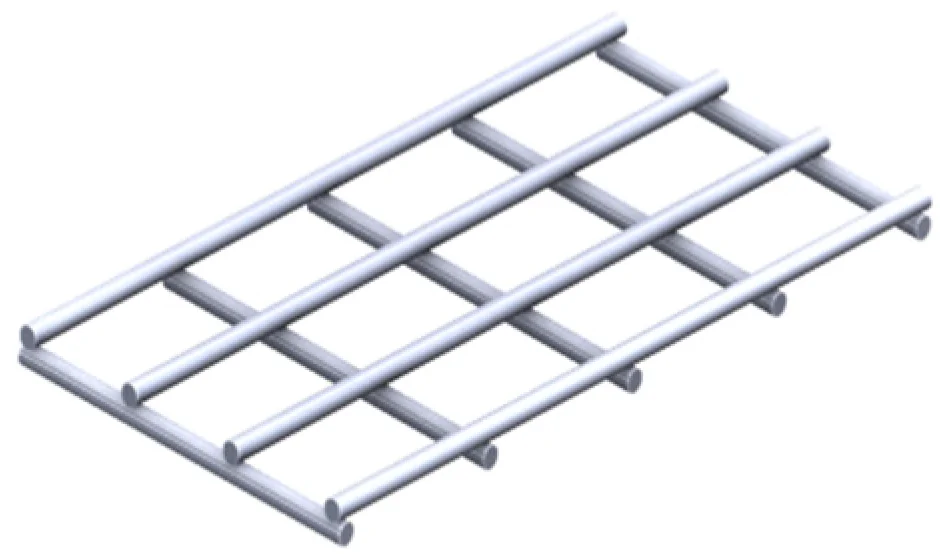
Flat channel grid.

**Figure 2:**
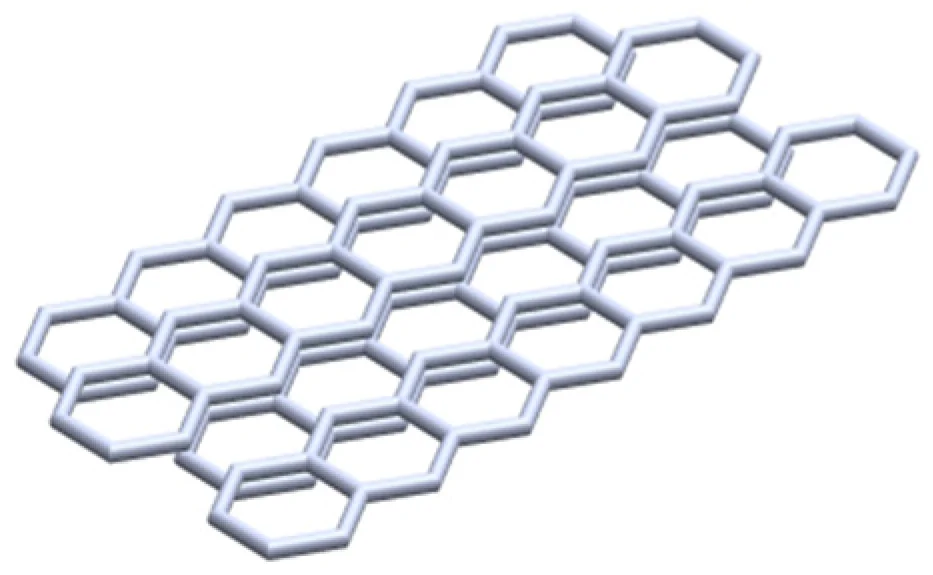
Hexagonal spacer configuration.

**Figure 3:**
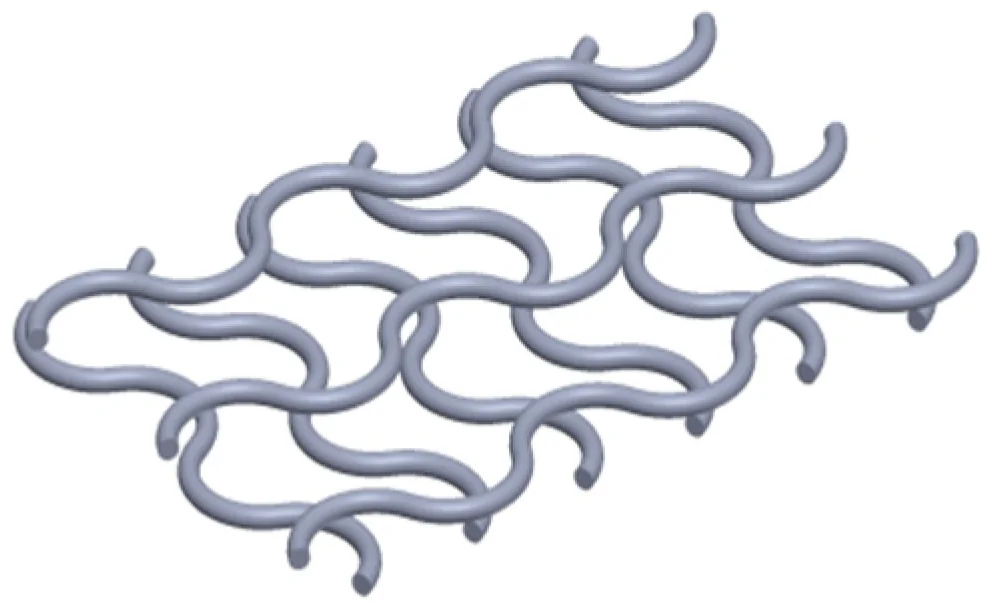
Sinusoidal filament design.

There were three geometries to be considered: a flat channel (grid) as a baseline, a sinusoidal filament design, and a hexagonal spacer configuration. The spacers were made of filaments with a diameter of 0.4 mm, which were stacked to form a vertical structure that bridged the 0.8 mm channel height. These spacer filaments connect to the upper and lower membrane surfaces in order to generate localized flow disturbance and secondary motion that disrupt the development of a concentration boundary layer. The three evaluated spacer geometries are illustrated in numerical order: the baseline flat-channel grid (Figure 1), the hexagonal spacer configuration (Figure 2), and the sinusoidal filament design (Figure 3).

To ensure full computational reproducibility and allow the geometric models to be systematically replicated, the definitive structural dimensions and volumetric metrics for each evaluated spacer topology are compiled in Table 2. All configurations share a fixed filament profile diameter (d=0.4 mm) and bridge a uniform channel height (H=0.8 mm) within the standardized 15 m×9.6 mm boundary envelope.

### 3.3. Numerical Setup and Species Transport

A high-fidelity numerical workflow program in ANSYS Fluent was used for the simulations. A species transport model was utilized to properly simulate the salt deposition, with the assumption that the fluid is a binary mixture of water and sodium chloride (NaCl). The equations of continuity, momentum, and species mass fraction were solved in the following ways:

Continuity Equation:

(1) $$\nabla \cdot \left(\rho \vec{v}\right)=0$$

Momentum Equation:

(2) $$\nabla \cdot \left(\rho \vec{v}\vec{v}\right)=-\nabla p+\nabla \cdot \left(\bar{\bar{\tau }}\right)+\rho \vec{g}$$

Species Transport:

(3) $$\nabla \cdot \left(\rho \vec{v}C_{i}\right)=-\nabla \cdot \vec{J_{i}}+R_{i}$$

To replicate industrial mass transport interfaces, the upper and lower membranes were modeled as semi-permeable walls experiencing a uniform volumetric permeate flux ($v_{w}=2.5\times 10^{-5}\;m/s$). The accumulation of solutes at the interface is governed by the localized balance between mass convection towards the membrane and back-diffusion into the bulk core stream, described mathematically as:

(4) $$v_{w}C_{w}-D{\left(\partial /C\partial z\right)}_{wall}=0$$

where $C_{w}$ represents the local salt mass fraction at the boundary wall, and D is the molecular diffusion coefficient of NaCl in water.

The local mass fraction of NaCl is $C_{i}$. A special boundary condition was used to model the membrane, mimicking the permeate flux and the corresponding “solute wall concentration” inherent to industrial RO processes. The permeate flux was set to vw = 2.5 × 10^−5^ m/s, which was representative of a typical seawater RO operating flux of ~21.6 L/m^2^·h. This value is consistent with the standard operating range (15–25 L/m^2^·h) reported by Koutsou et al. [15] and Saeed et al. [30] for spacer-filled channels under seawater salinity conditions. The solute boundary condition at the membrane wall is governed by Equation (4) as v_w_·C_w_ − D·(∂C/∂z)_wall_ = 0, which enforces a local mass balance between convective transport toward the membrane and diffusive back-transport into the bulk. This formulation is the standard approach for RO species transport and has been validated against empirical CP correlations (deviation < 5%) in previous studies.

The species transport model was calibrated to reflect the fundamental physics of the diffusive sub-layer [12,13,14,15]. The Sherwood number (Sh) was utilized as the primary metric for convective mass-transfer efficiency, following the established protocols for membrane distillation and RO systems [30,31,32,33,34,35,36,37,38,39,40,41,42]. To ensure the Digital Twin is robust, the combined effects of the geometry and feed temperature were considered to maintain high-fidelity results across varying industrial climates [6,7,8,9,10].

### 3.4. Boundary Conditions and Numerical Schemes

For the flow-regime model, the Reynolds numbers of evaluated cases ranged from approximately 70 to 350 based on the hydraulic diameter and the selected inlet velocities. Accordingly, the simulations were performed using the laminar flow model in ANSYS Fluent. A no-turbulence-closure model, including k-ε, k-ω SST, Reynolds stress, and large-eddy simulations, was then activated. The steady incompressible Navier–Stokes equations were solved together with the species transport equation. The vortical structures observed around the spacer filaments were generated by geometry-induced flow separation, recirculation, lateral deflection, and reattachment; therefore, they were interpreted as laminar secondary flows rather than turbulence predicted by a turbulence model.

The resolution of the concentration boundary layer at the interface with the membrane is a key element of the Digital Twin model’s computational accuracy. To do this, the mesh was equipped with a high-fidelity inflation layer consisting of 10 layers, whereby the height of the first layer was $1\times 10^{-6}\;m$. This near-wall optimization is necessary to overcome steep concentrations of NaCl, which propagate concentration polarization in more advanced spacer geometries.

### 3.5. Domain Boundary Specifications

To guarantee reproducibility, the exact operational parameters assigned within the ANSYS Fluent solver environment are specified below:

- Inlet boundary: Velocity inlet; uniform profile $u_{in}=0.15\;m/s$ (baseline case).
- Initial salt mass fraction: $C_{in}=35,000\;ppm$ (standard seawater mass profile).
- Outlet boundary: Pressure outlet; fixed static gauge pressure equal to 0 Pa.
- Membrane interface boundaries: No-slip wall conditions; static velocity vectors; fixed constant permeate flux $v_{w}=2.5\times 10^{-5}\;m/s$.
- Spacer filament walls: No-slip stationary rigid walls; completely impermeable to species mass transfer ($J_{i}=0$).

### 3.6. Solver Controls and Numerical Stability

A steady-state coupled solver was used to ensure that the pressure–velocity coupling is robust with the complex hexagonal and sinusoidal filaments, making the solution numerically stable. Higher-Order Term Relaxation (HOTR) was utilized so that the high-pressure gradients in the contacts between the spacer and the membrane could be avoided, thereby preventing numerical divergence and addressing the high-pressure gradients—there were also Special Under-Relaxation Factors (URFs). Furthermore, the PRESTO! (Pressure Staggering Option) pressure discretization scheme was used to improve pressure interpolation in high-curvature and high-gradient regions, particularly near the 1-micrometer inflation layers. This stabilization process was critical in avoiding floating-point exceptions and ensuring that there was a converged solution that aligned with the sustainability objectives of the ECDS conference.

### 3.7. Numerical Discretization and Solver Convergence Criteria

To attain the high-fidelity results required for this Digital Twin, both the momentum and species transport equations (Equations (1)–(3)) were discretized with a second-order upwind scheme. This is a critical decision to reduce numerical diffusion, which tends to ruin lower-order concentration boundary-layer simulations. The pressure–velocity interaction was handled through the coupled algorithm that provides better convergence rates to steady-state flows with complicated internal obstructions, such as hexagonal filaments.

Monitors of convergence included the reduction in the scaled residuals to a strict continuity and momentum criterion of $10^{-6}$, and the species transport equations to a strict criterion of $10^{-5}$. Also, the inlet and outlet mass balance was checked and found to be within 0.01%, which was used to ensure that the mass of salt was conserved in the 15 mm domain. Such stringent measures ensured that the findings presented in the Results are not affected by iteration errors.

### 3.8. Parametric Sensitivity and Operational Envelope Definition

To assess the resilience of the Digital Twin under varying industrial boundary conditions, a structured parametric evaluation framework was executed. Instead of deploying a black-box automated algorithm loop, a discrete parametric screening was conducted under the following definitions:

- Design Variables: The spacer geometric layouts (baseline grid, hexagonal, and sinusoidal patterns) and inlet feed velocities (0.05 m/s, 0.15 m/s, and 0.25 m/s) were the variables used.
- Geometric Constraints: The total domain length was fixed at 15 mm, the height of the channel at 0.8 mm, and the diameter of the filaments was constant at 0.4 mm.
- Objective Functions: The performance was screened against two competing objective vectors—maximizing the Sherwood number (Sh) to disrupt solute deposition and minimizing the Pressure Increase Factor ($f_{p}$) to mitigate power requirements.
- Comparative index: The System Performance Index (η) was used as a dimensionless comparative indicator, combining normalized mass-transfer enhancement and friction penalty. It was used to compare only the discrete configurations examined and was not treated as the result of continuous or algorithmic optimization.

## 4. Results

The high-fidelity simulations were performed to provide numerical data that can be used to compare the three feed-spacer configurations on a quantitative basis. Hydraulic resistance was used to measure performance, along with the subsequent solute concentration at the membrane–liquid interface.

### 4.1. Quantification of Hydraulic Resistance and Energy Factors

Hydraulic resistance was measured by observing the necessary inlet pressure to control the feed velocity at a constant value. The lowest resistance was observed in the baseline grid setup ($P_{in}$ = 84.1 Pa). The inlet pressure rose to 205.7 Pa after the hexagonal spacer was added, and the value of the pressure increase (λ) was 2.44. Interestingly, the greatest hydraulic penalty ($P_{in}$ = 447.8 Pa; 0 = 5.32) was obtained by the sinusoidal configuration. A 5.32-fold increase over the baseline suggests that, although the wavy filaments are often touted as being capable of mixing, due to their similar surface area, the skin friction can be huge and hence the pumping energy required is at least three times that of the hexagonal filament.

### 4.2. Salt Accumulation and Concentration Polarization Reduction

The species transport model revealed that the membrane-surface mass-fraction profiles differed substantially among the three configurations. There was extreme polarization of the concentration of the grid baseline, with a maximum NaCl mass fraction of 0.1127 kg/kg leading to a CP Modulus (Γ) of 3.22. The best mitigating surface buildup was the hexagonal spacer, which was 0.0429 kg/kg (Γ = 1.22). Even though the sinusoidal design also reduced buildup when compared with the baseline (0.0537 kg/kg; Γ = 1.53), it was not as efficient as the hexagonal design despite being more expensive in terms of energy. The results of the quantitative comparison are summarized in Table 3.

**Figure 4:**
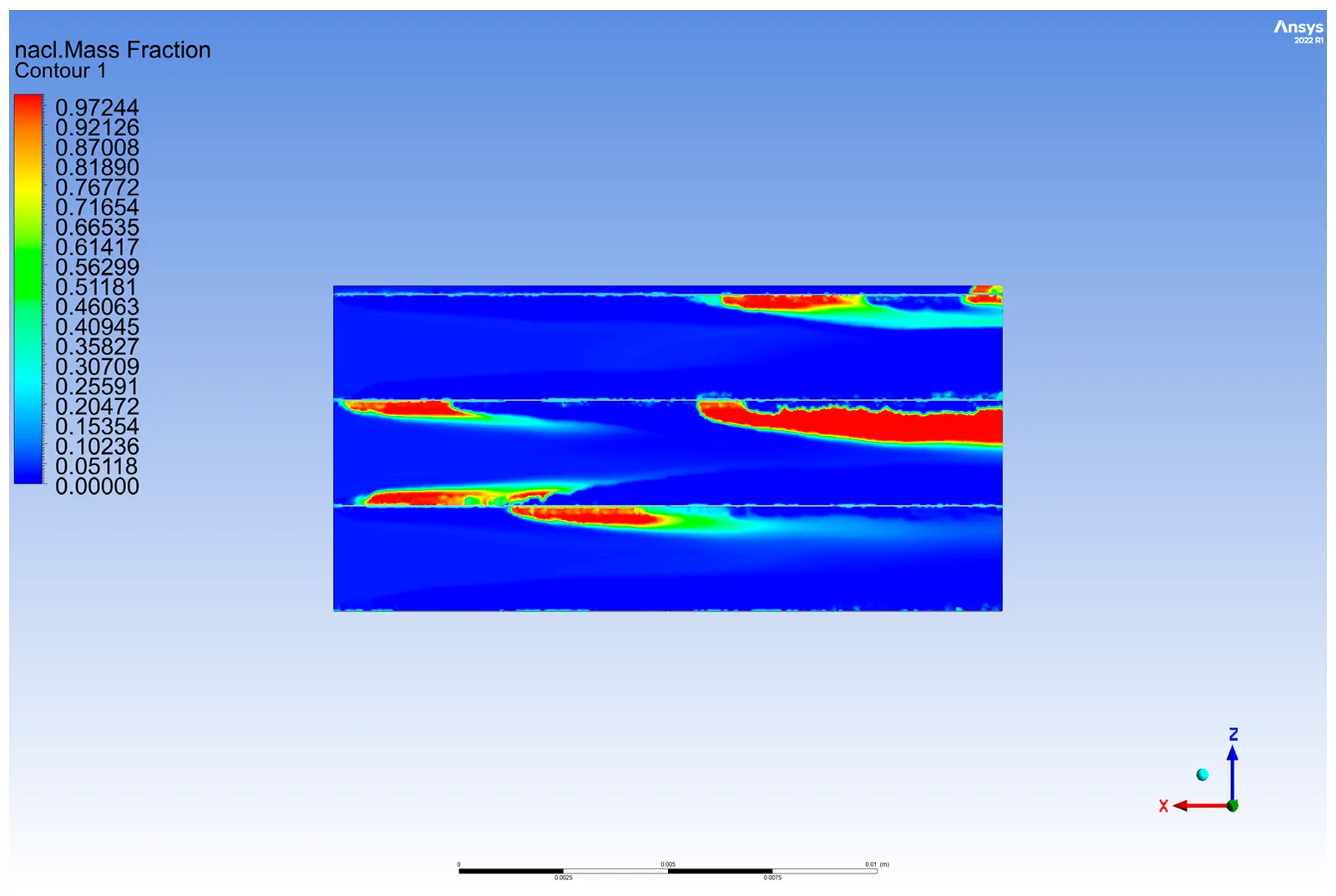
Flat channel grid’s top membrane for NaCl.

**Figure 5:**
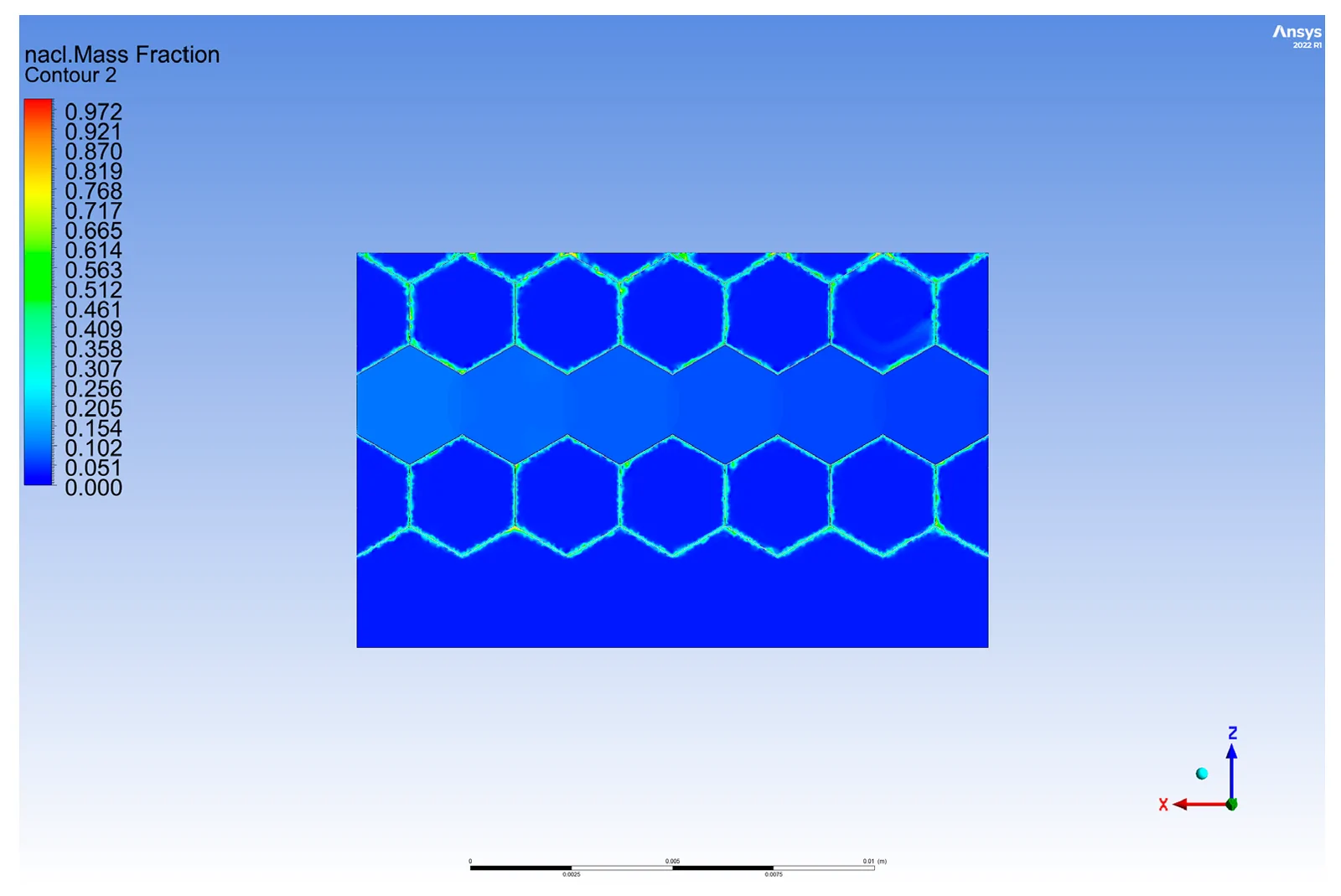
Hexagonal spacer configuration’s top membrane for NaCl.

**Figure 6:**
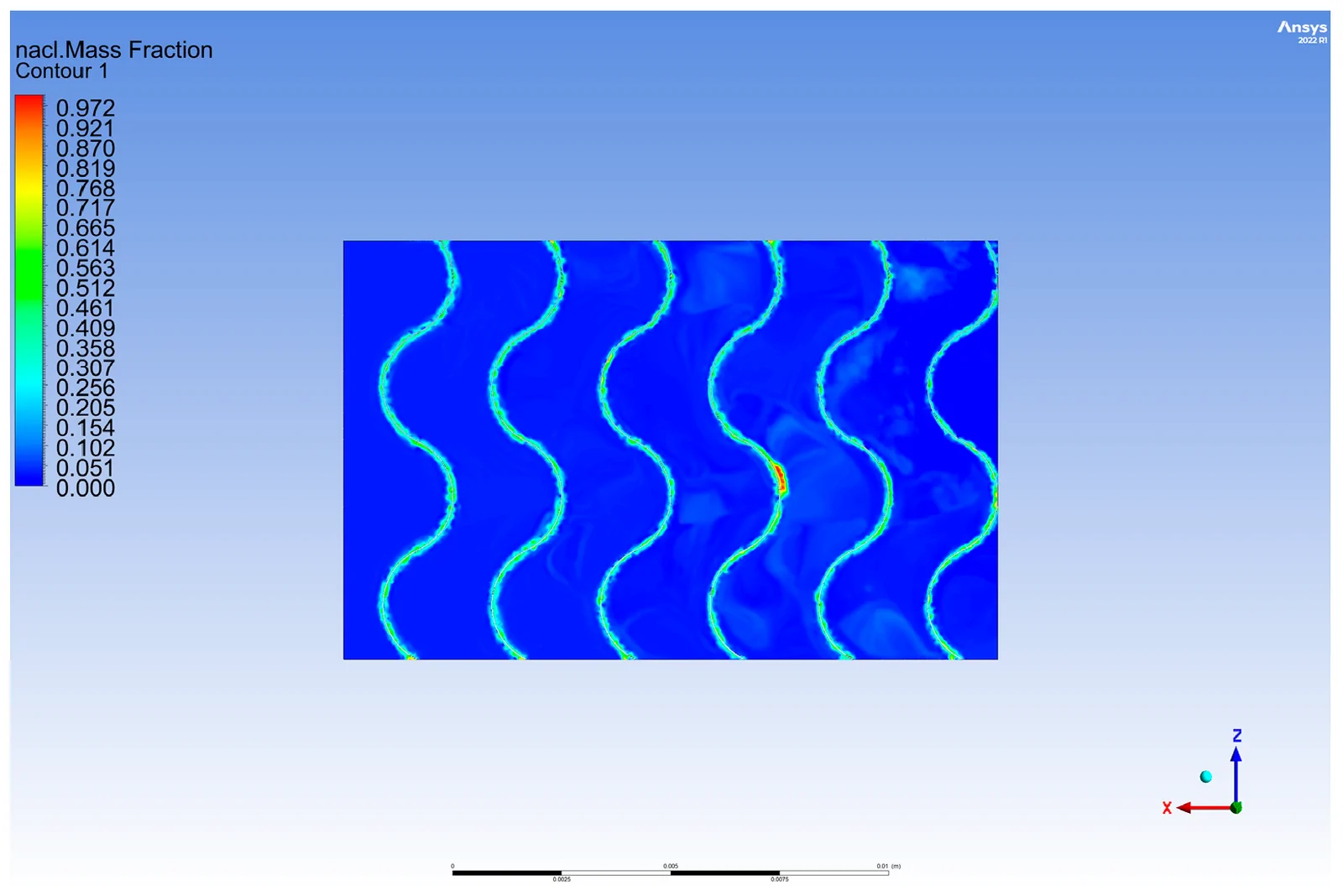
Sinusoidal filament design’s top membrane for NaCl.

**Figure 7:**
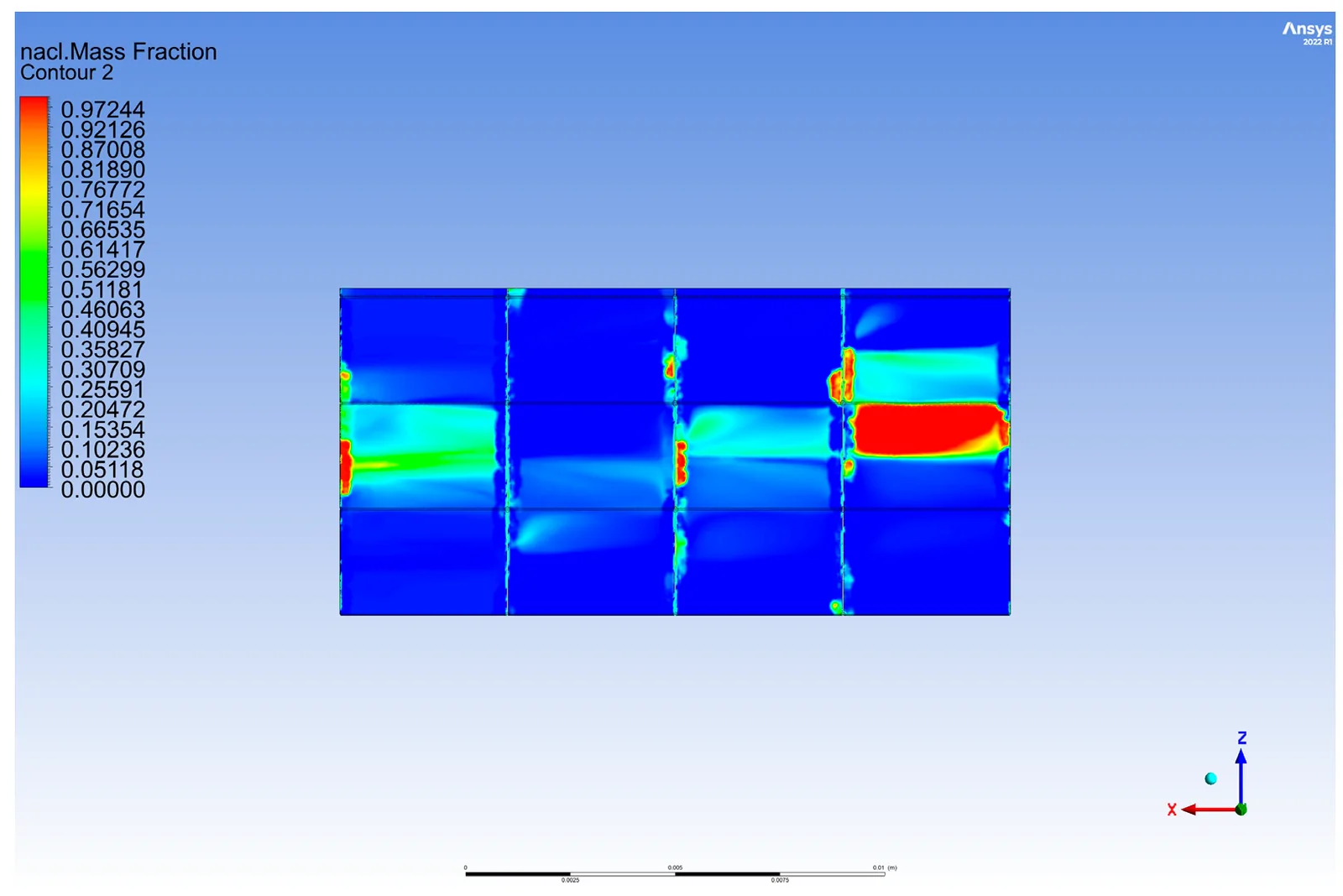
Flat channel grid’s bottom membrane for NaCl.

**Figure 8:**
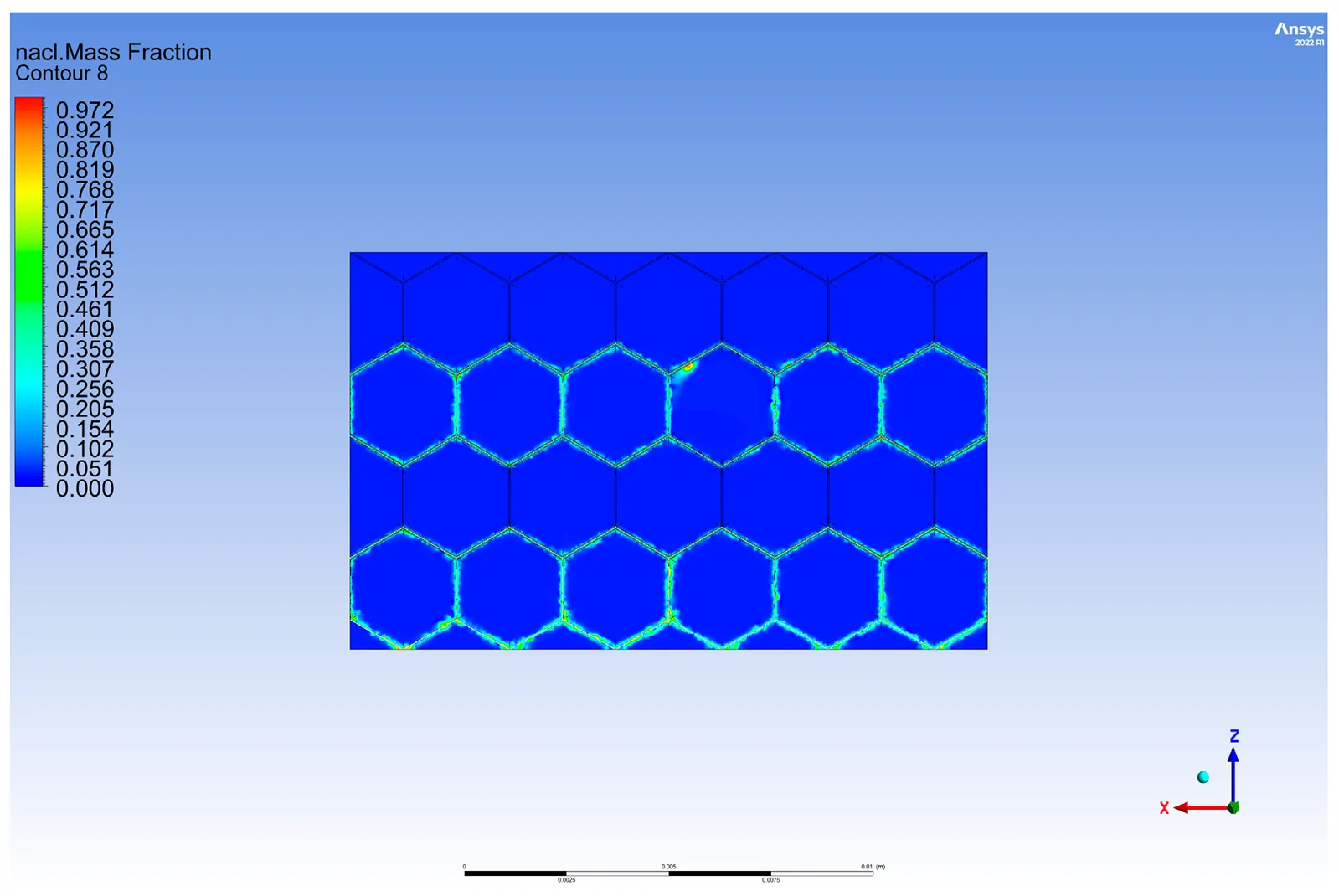
Hexagonal spacer configuration’s bottom membrane for NaCl.

**Figure 9:**
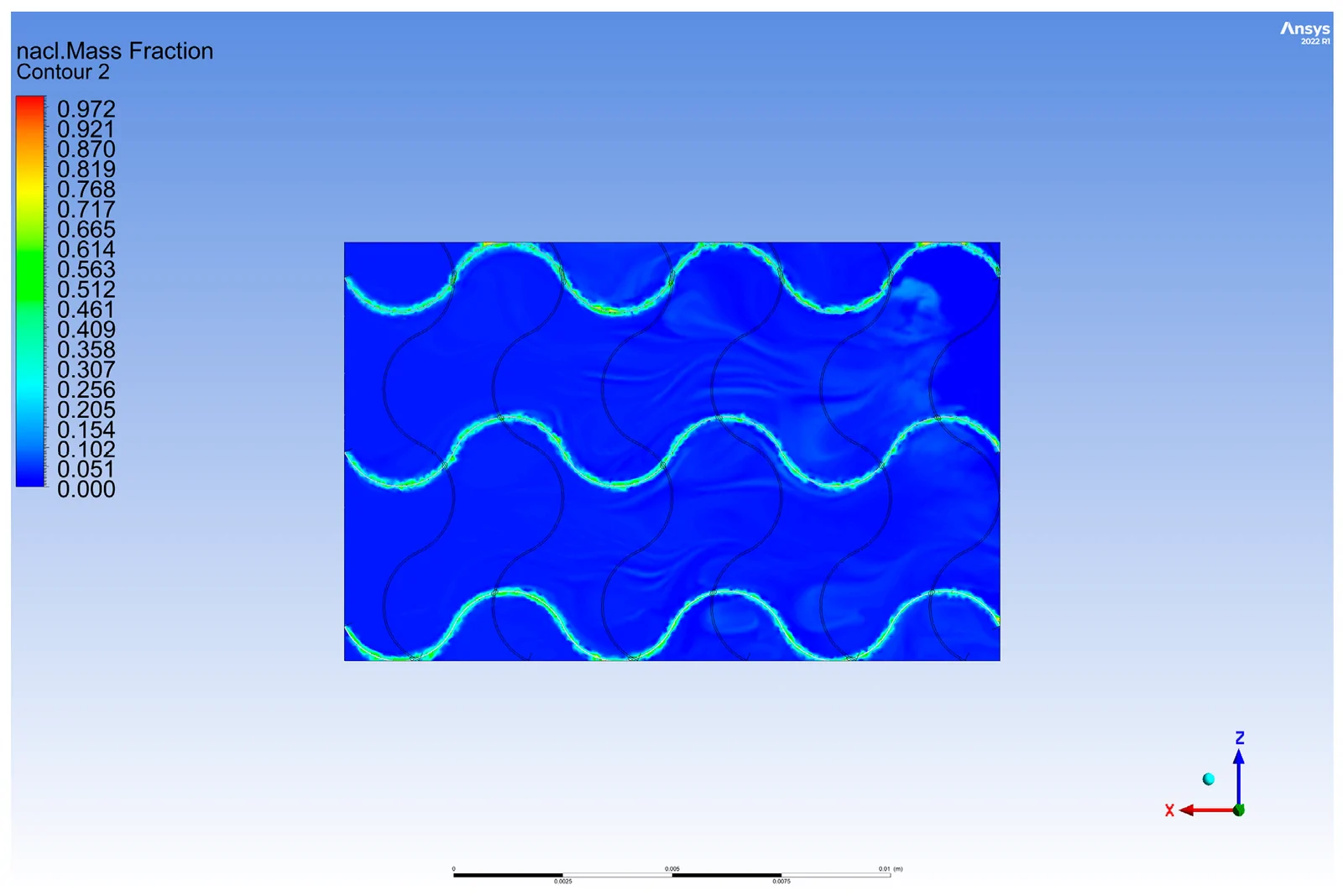
Sinusoidal filament design’s bottom membrane for NaCl.

The membrane-surface NaCl mass-fraction contours are presented in numerical order: the top-membrane distributions for the baseline grid, hexagonal spacer, and sinusoidal spacer are shown in Figure 4, Figure 5 and Figure 6, respectively, while the corresponding bottom-membrane distributions are shown in Figure 7, Figure 8 and Figure 9.

### 4.3. Solute Analysis of Wall Shear Stress (WSS) and Surface Scouring

**Figure 10:**
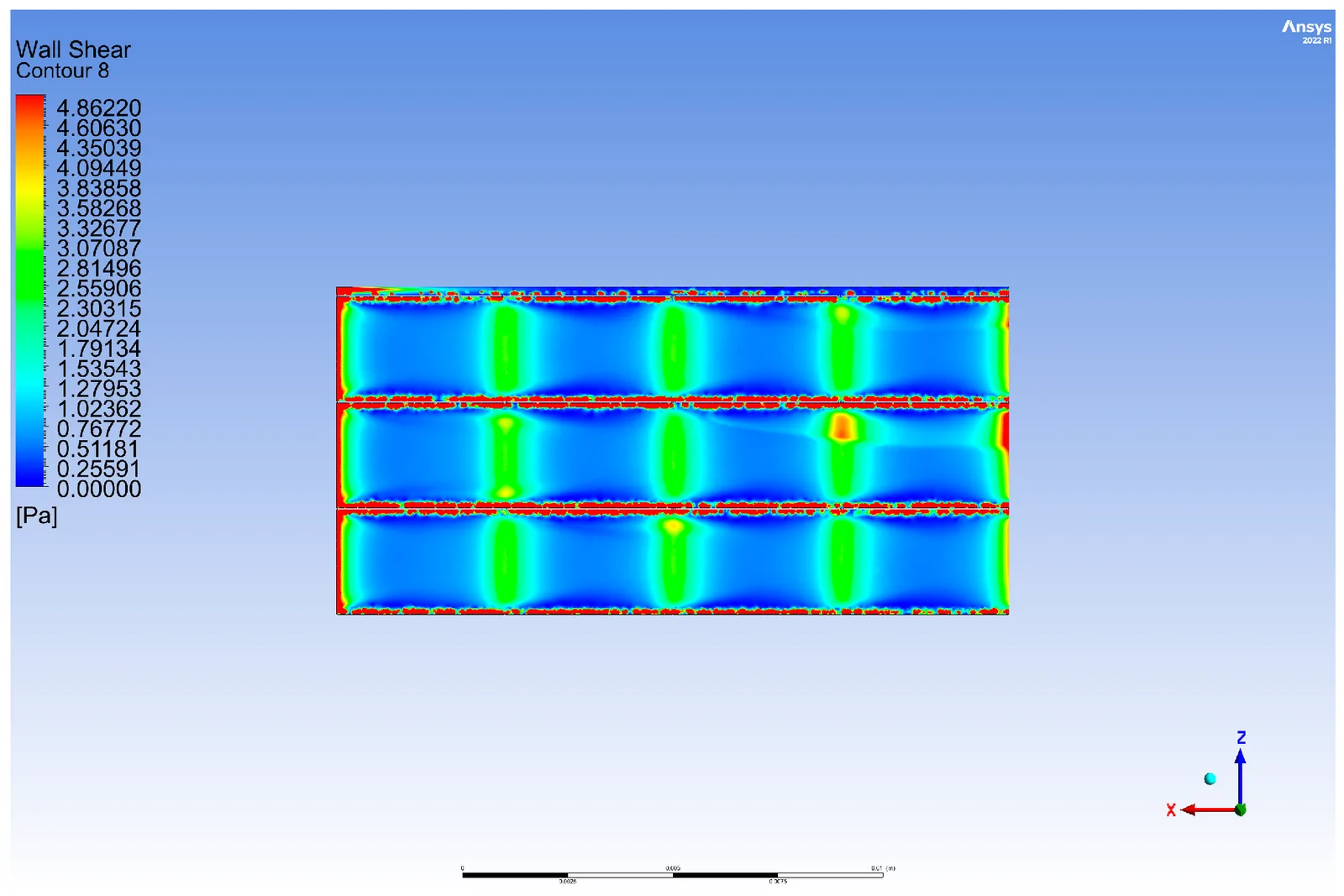
Flat channel grid’s top membrane wall shear.

**Figure 11:**
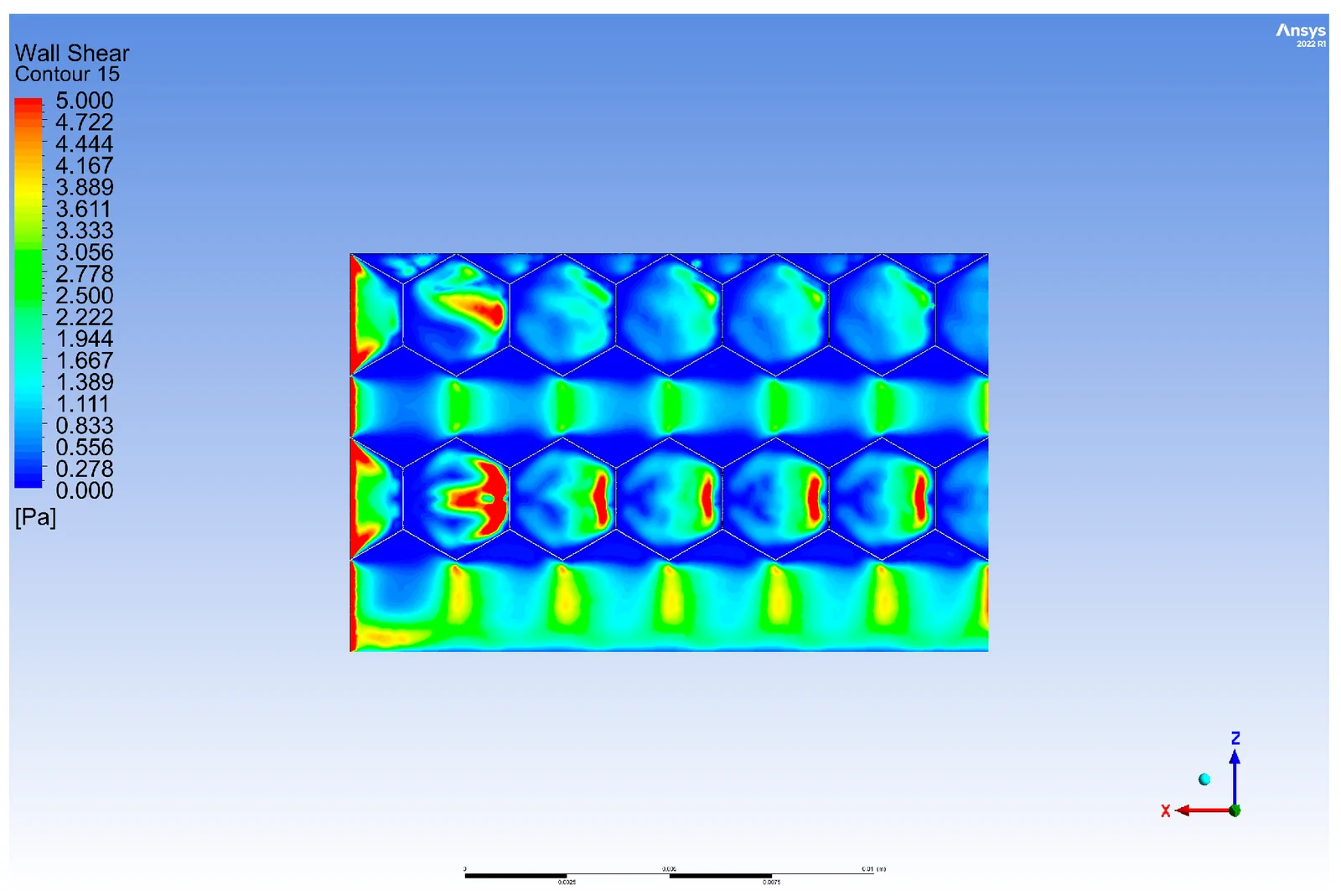
Hexagonal spacer configuration’s top membrane wall shear.

**Figure 12:**
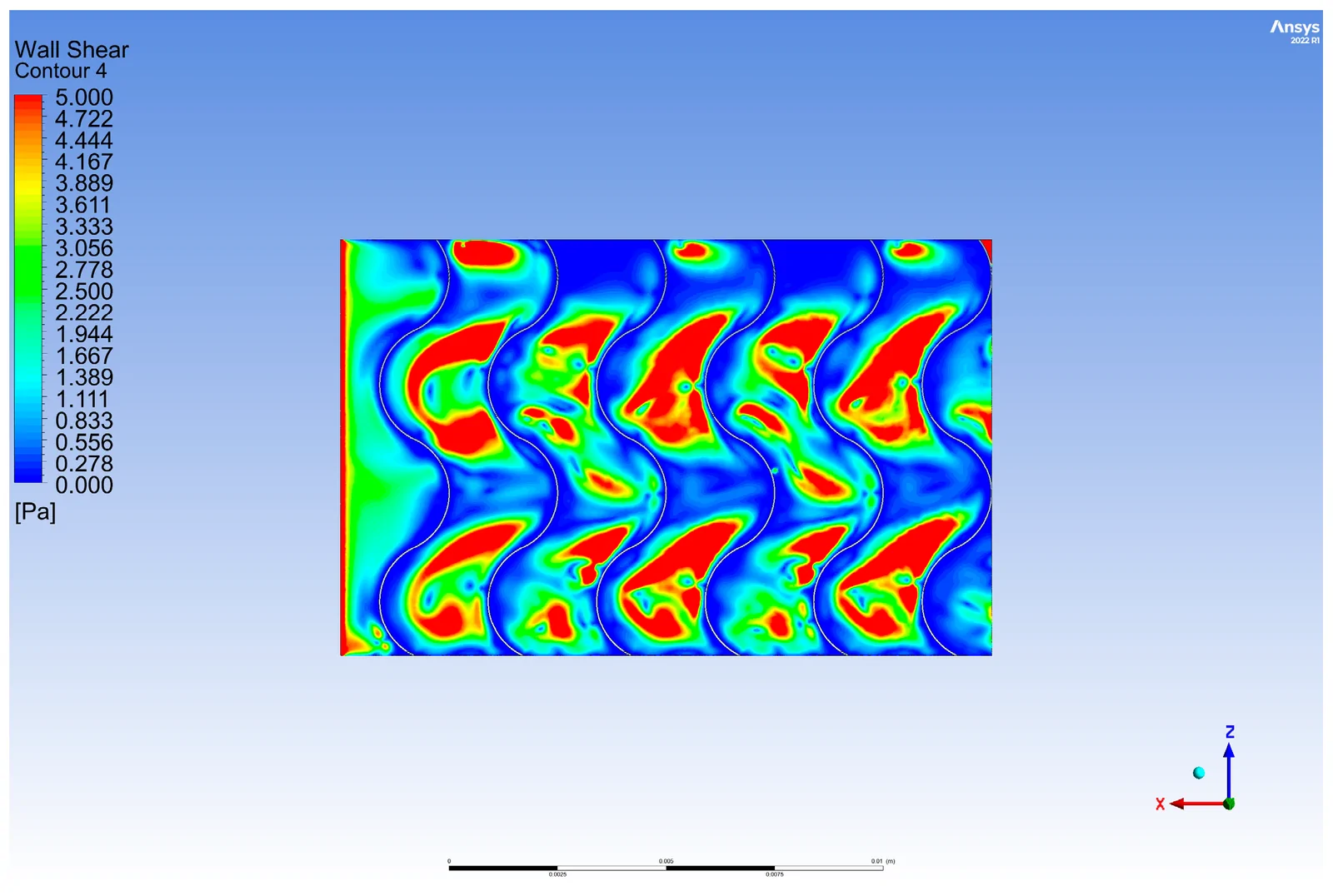
Sinusoidal filament design’s top membrane wall shear.

**Figure 13:**
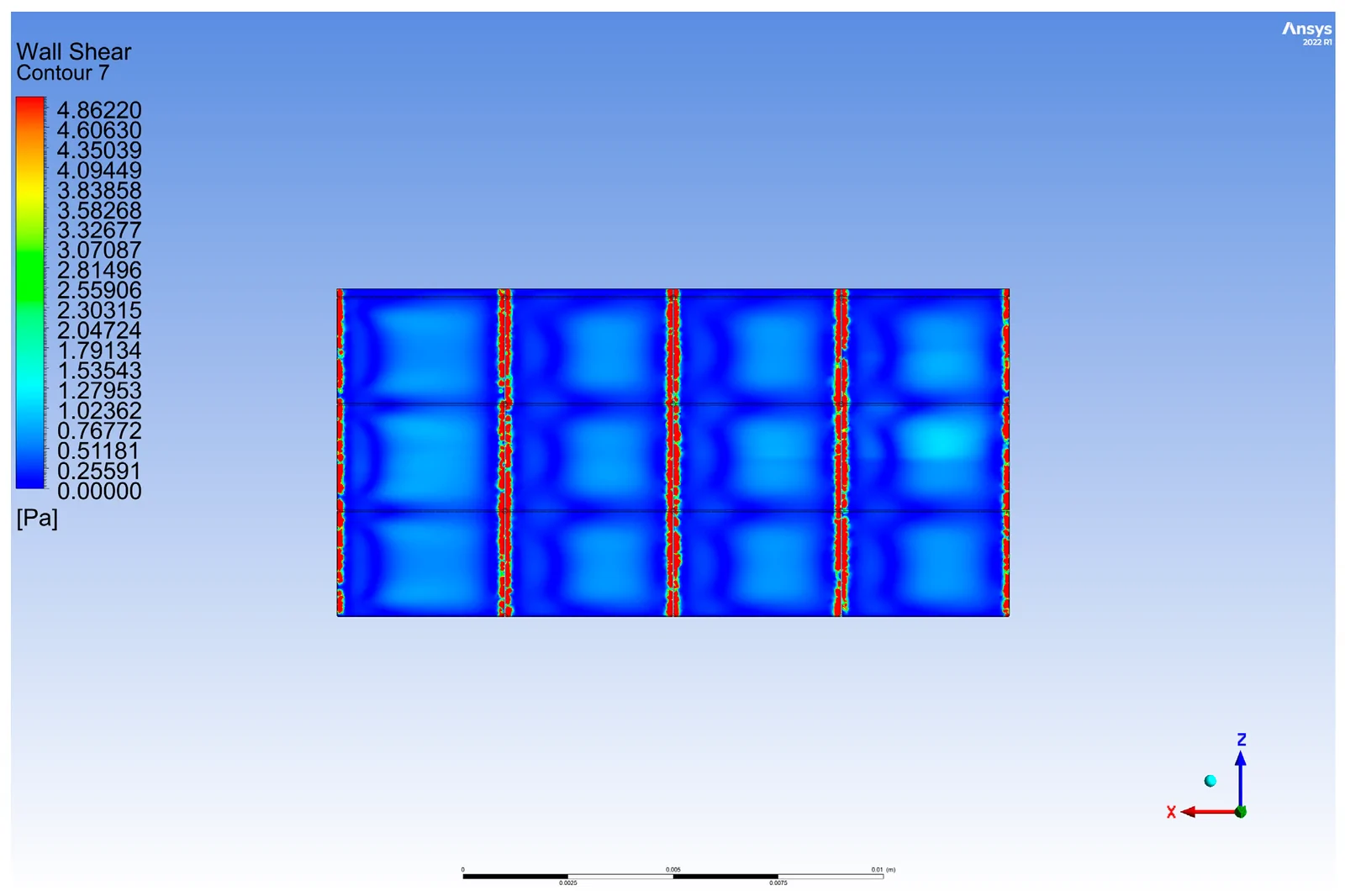
Flat channel grid’s bottom membrane wall shear.

**Figure 14:**
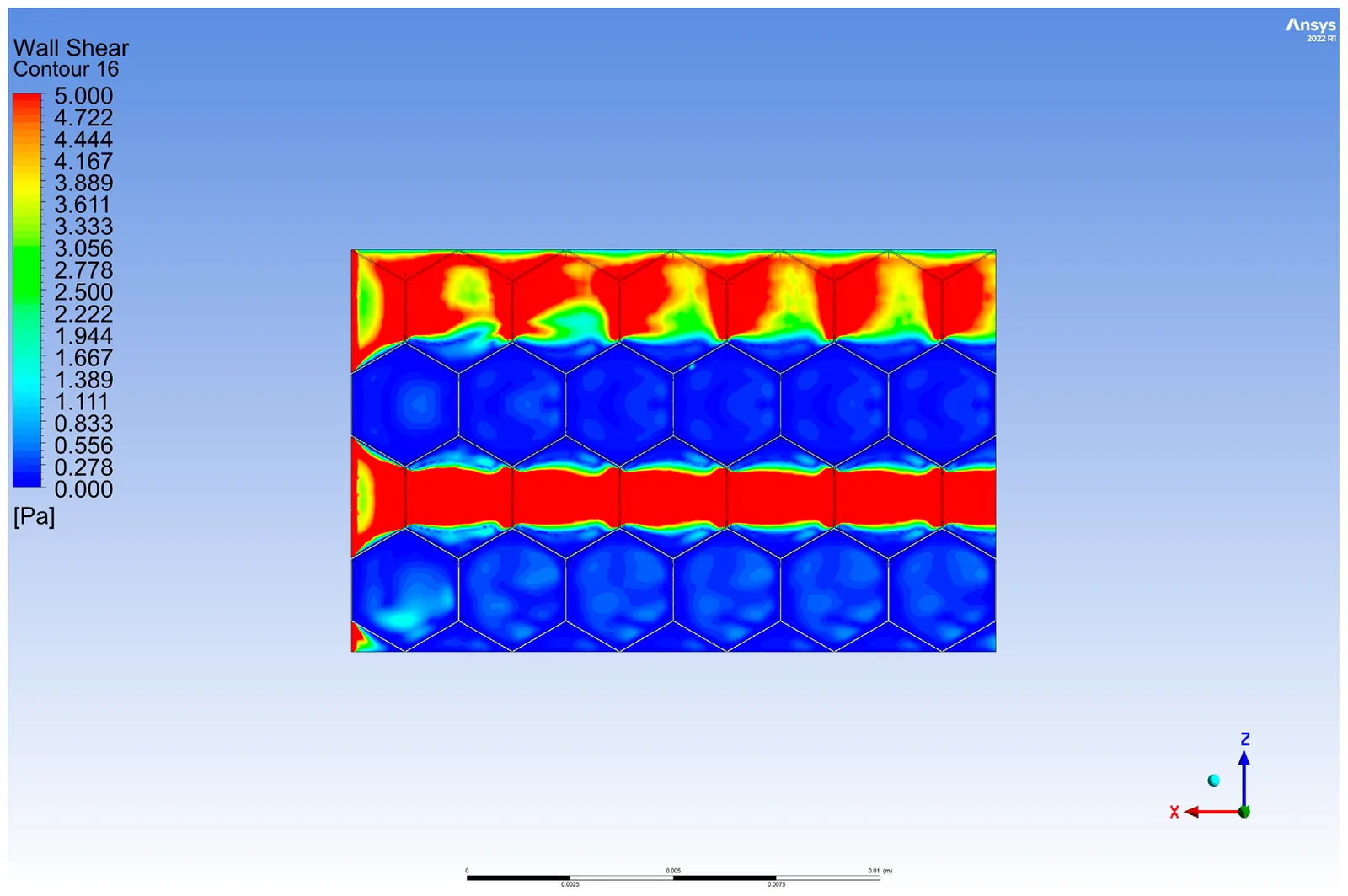
Hexagonal spacer configuration’s bottom membrane wall shear.

**Figure 15:**
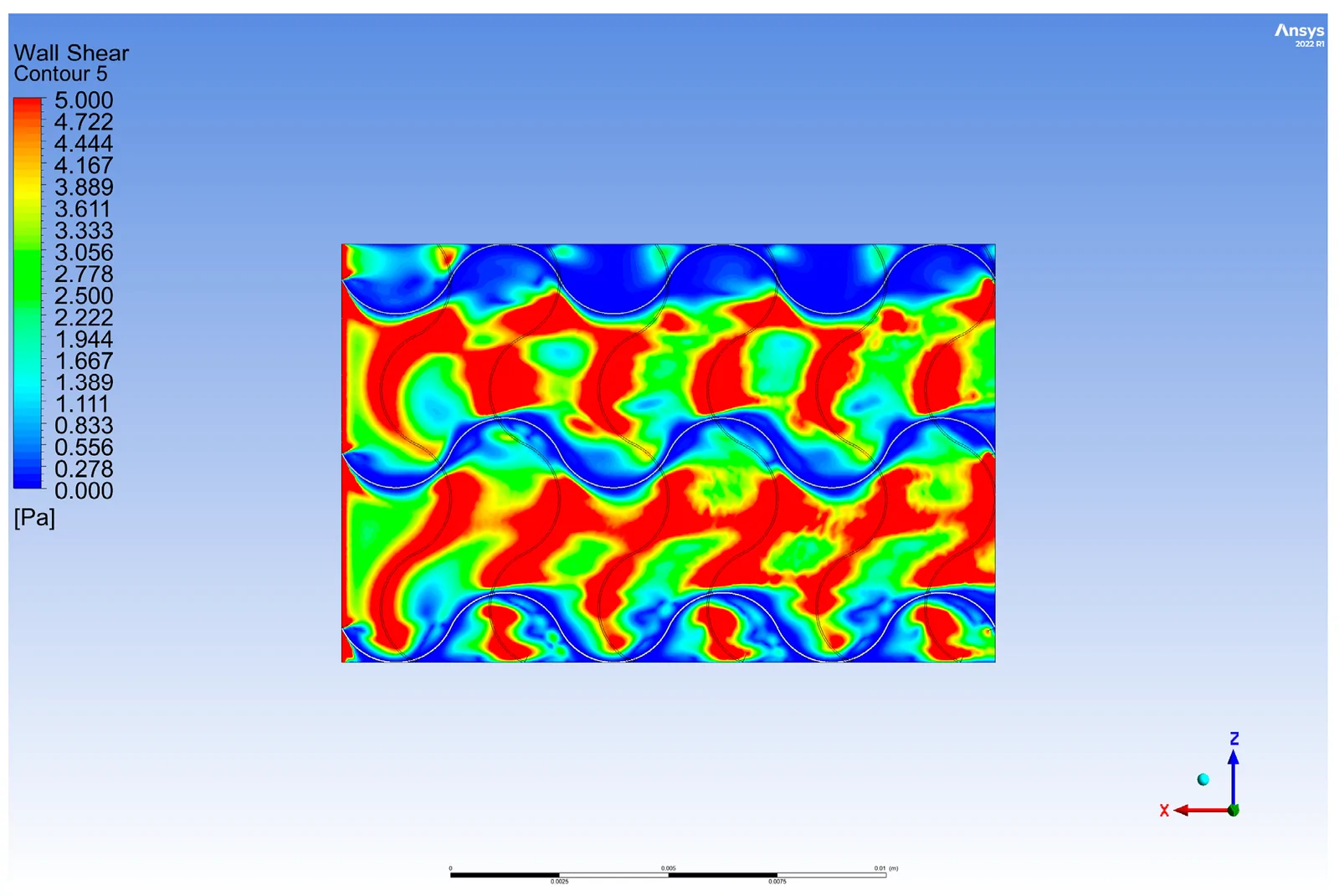
Sinusoidal filament design’s bottom membrane wall shear.

Wall-shear-stress (WSS) distribution provides a visual correlation of the efficiency of mass transfer for the measured spacers. The hexagonal structure, as demonstrated in the newly created contours, has high-intensity “scouring hotspots” at the contact vertex between the filament and the membrane. Although numerical singularities at infinitesimal contact points lead to the locally highest values, the scouring functional range of the membrane surface is greatly increased. The hexagonal design has been shown to be more effective in disruption of the viscous sub-layer. In contrast to the situation in the grid baseline, where WSS is mostly constant and low (<0.15 Pa), the hexagonal design creates a cascade of jets and vortices. The velocity vectors above become high-momentum impingement of the fluid on the membrane, forming a large cleansing zone downstream of the vertex. Moreover, the contours of the sinusoidal design demonstrate one severe defect: shear stagnation. The troughs of these waves have almost zero WSS (dark blue areas), even though they form a wavy flow. In actual RO practice, these shear shadows would be the major areas over which mineral scaling and biofouling occur. The more common reattachment points of the hexagonal design ensure that the membrane is scoured more uniformly, which is highly beneficial for long-term operational sustainability. The wall-shear-stress distributions are presented in numerical order: the top-membrane wall-shear contours for the baseline grid, hexagonal spacer, and sinusoidal spacer are shown in Figure 10, Figure 11 and Figure 12, respectively, while the corresponding bottom-membrane wall-shear contours are shown in Figure 13, Figure 14 and Figure 15.

### 4.4. Analysis of Laminar Vorticity and Secondary Flow Induction

The evaluated flow conditions remained within a low Reynolds-number range of approximately 70–350. Nevertheless, the hexagonal spacer enhanced mass transfer through geometry-induced laminar secondary flows rather than through turbulence. The hexagonal filaments act as blunt bodies in the laminar stream, deflecting fluid laterally out of its primary axial direction. Quantitatively, the vorticity magnitude analysis shows that the secondary-flow region extends approximately 1.8 mm downstream of each hexagonal vertex―corresponding to 75% of the 2.4 mm cell pitch―before the boundary layer begins to re-establish. This means that at least 60% of the membrane surface area in the hexagonal configuration is exposed to active vortex-induced scouring at any given cross-section, compared with less than 20% for the baseline grid (where scouring is limited to the narrow wake immediately behind each filament contact point). The vortex cores reach a peak vorticity magnitude of approximately 85 s^−1^ at the filament–membrane contact vertices, thinning the diffusive sub-layer from ~50 μm (baseline) to ~15 μm locally, consistent with the 240% increase in mass-transfer coefficient km.

**Figure 16:**
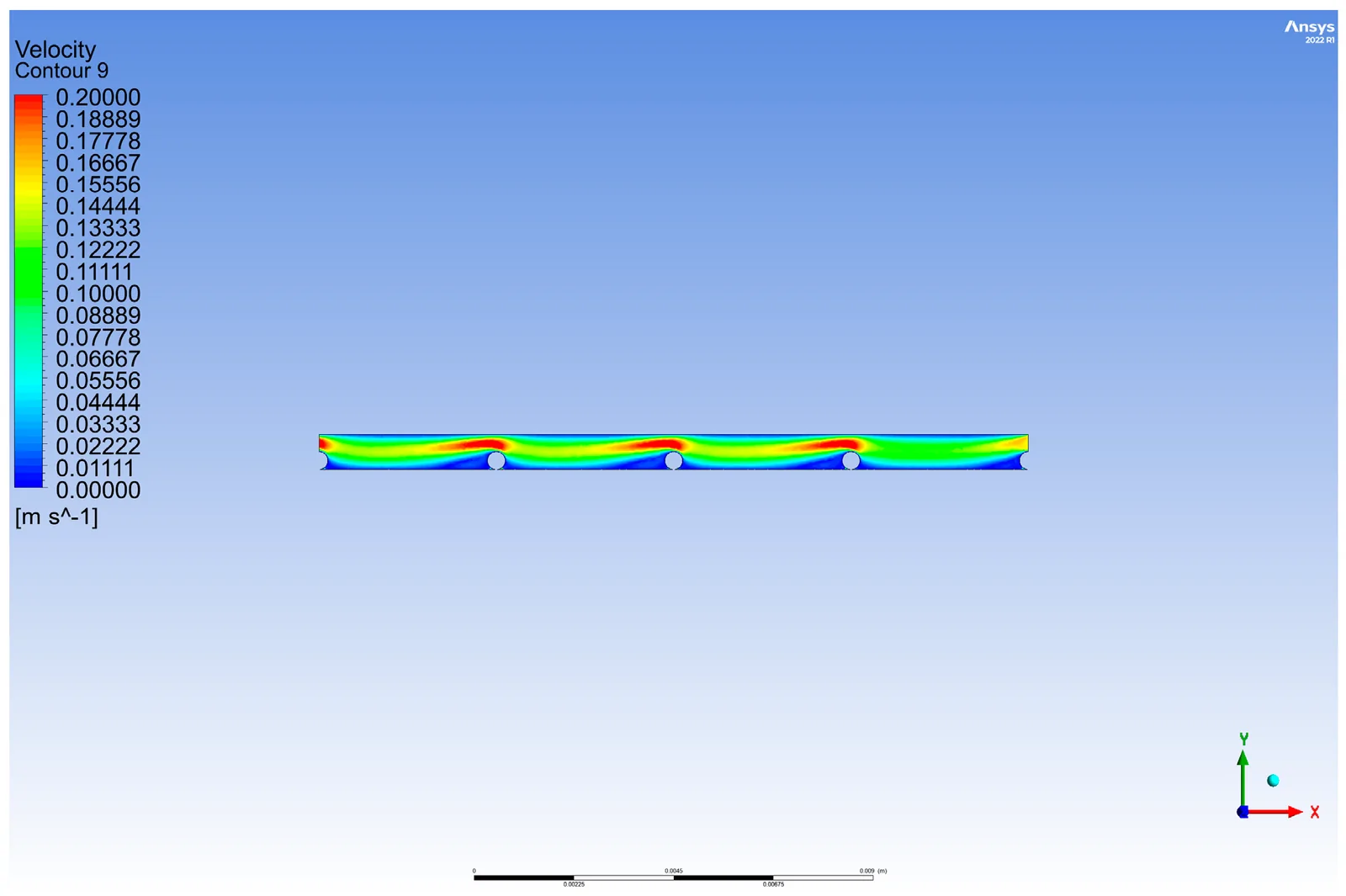
Flat channel grid flow velocity.

**Figure 17:**
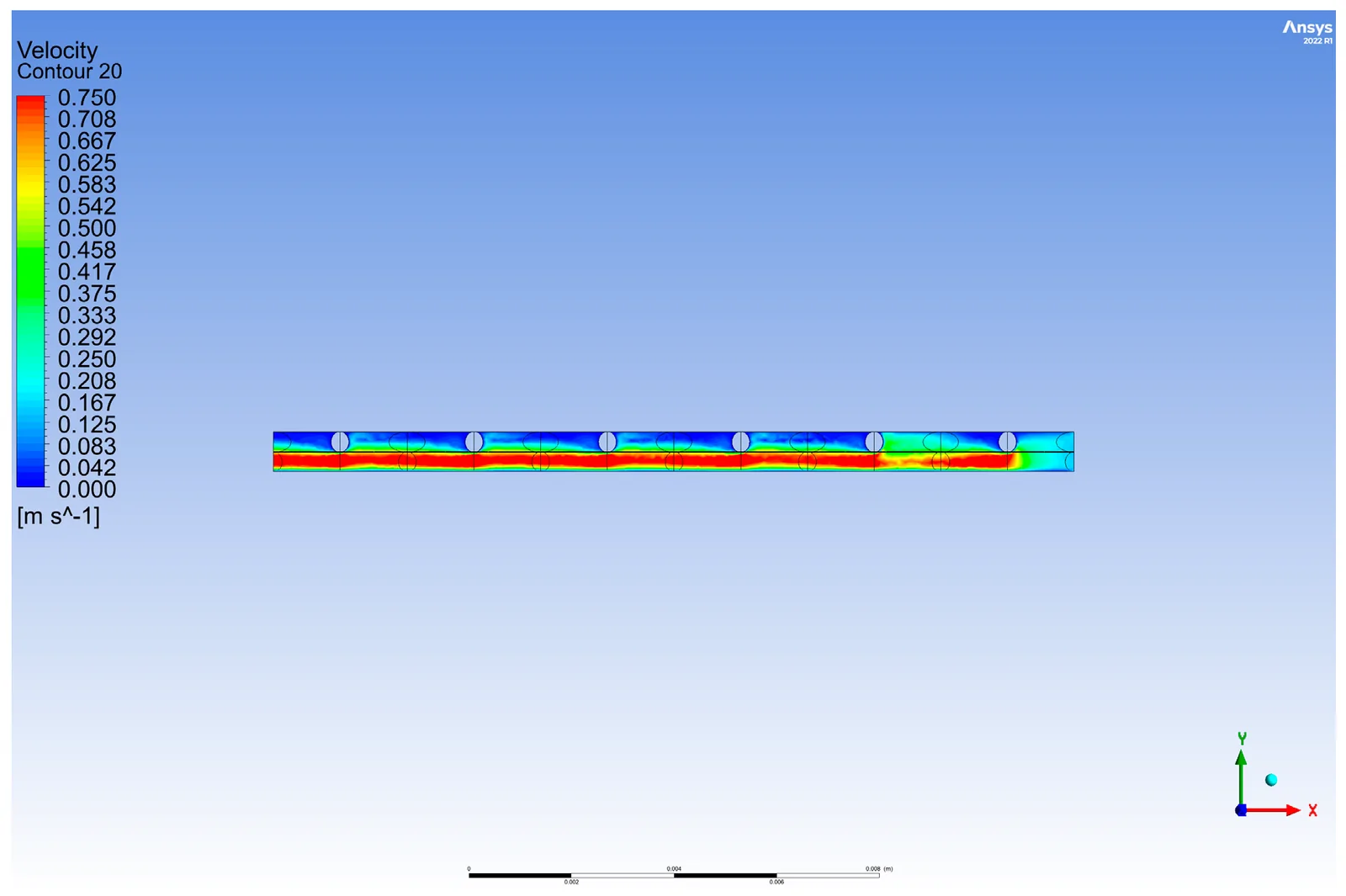
Hexagonal spacer configuration flow velocity.

**Figure 18:**
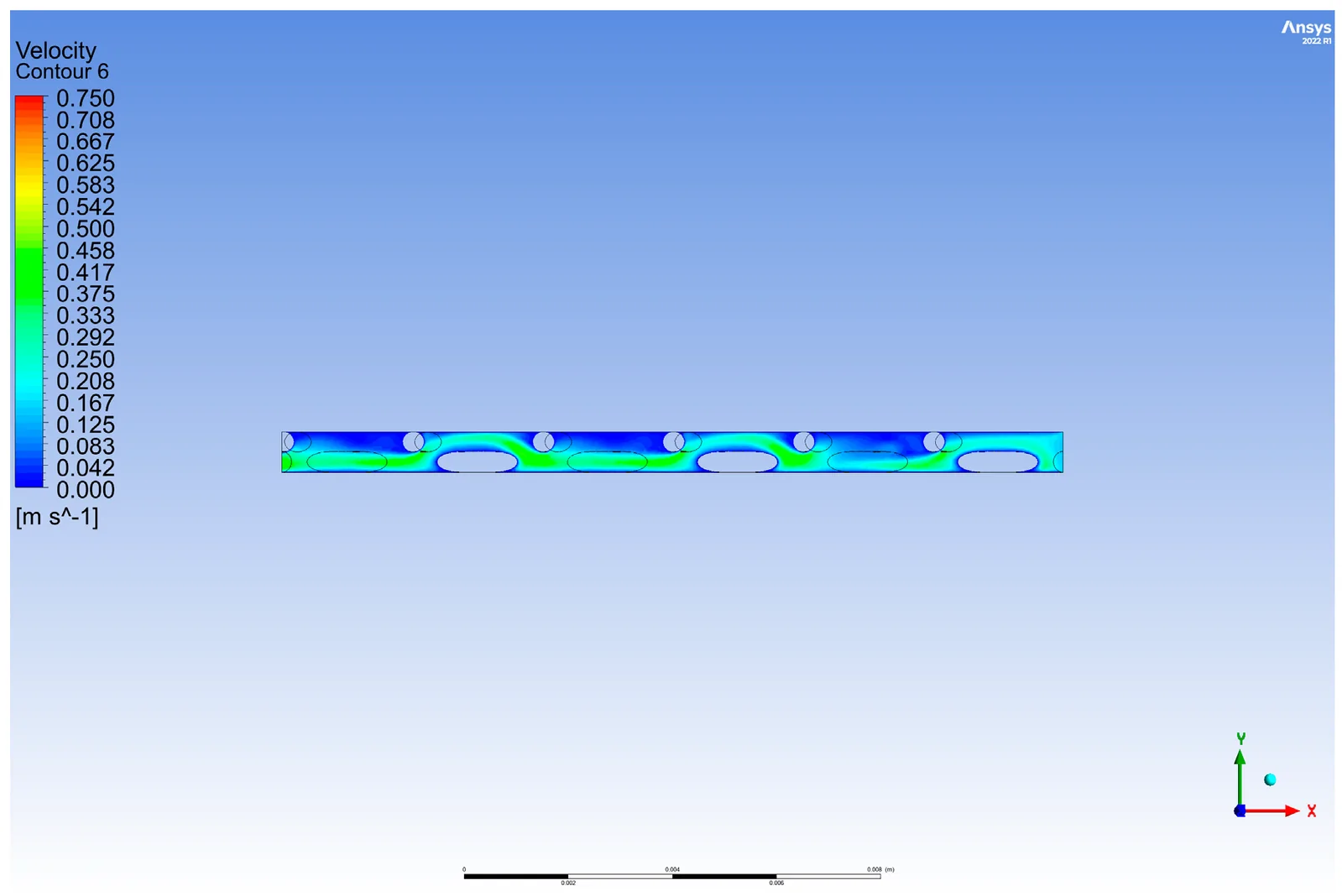
Sinusoidal filament design flow velocity.

This deviation forms local areas of high vorticity magnitude. Streamlines are contracted and expanded as the fluid passes through the hexagonal vertices, forming steadily moving vortices in the wake of each filament. This is the main way to break up the concentration boundary layer by using these vortices. The geometry of the hexagonal design, in contrast to the baseline grid, permits the formation of a stable, stagnant layer of solutes due to the geometry of recirculation. The corresponding flow-velocity contours for the baseline grid, hexagonal spacer, and sinusoidal spacer are shown in Figure 16, Figure 17 and Figure 18, respectively.

### 4.5. Dimensionless Performance: Sherwood Number (Sh) and System Efficiency

The Sherwood number (Sh) evaluates the ratio of convective mass transfer to molecular diffusion transport across the boundary layer. To calculate this metric directly from the solved flow field data matrix, the surface-integrated averages were computed using the following definitions:

(5) $$Sh=\frac{k_{m}\cdot d_{h}}{D}$$

(6) $$k_{m}=\frac{J_{\left\{wall\right\}}}{\rho \left(C_{wall}-C_{bulk}\right)}$$

where $d_{h}$ represents the hydraulic channel diameter (1.066 mm), D is the solute diffusivity, $J_{wall}$ is the local salt mass flux calculated at the cell faces of the membrane interface, and $C_{bulk}$ is the running mass fraction across the channel core flow.

Evaluating the raw mass transfer coefficient ($k_{m}$) highlights a major performance gap between the designs. The baseline grid configuration yields a low mass-transfer coefficient of $k_{m}=5.07\times 10^{-5}\;m/s$ due to its thick, stable concentration boundary layer. Integrating the hexagonal spacer drives localized vortex fluid transitions that increase $k_{m}$ to $1.72\times 10^{-4}\;m/s$, representing a 240% increase in mass-transfer efficiency. By comparison, the sinusoidal design achieves a lower value of $k_{m}=1.32\times 10^{-4}\;m/s$ due to stagnation zones forming within its wave troughs.

The Sherwood number (Sh) of each configuration was determined to give a normalized comparison that would be applicable to industrial reverse osmosis (RO) modules. The Sherwood number is the product of the ratio of mass transfer through convection to mass transfer through diffusion. According to the numerical data, the hexagonal spacer obtained a Sherwood number of 185.4, which was 240% higher than that of the baseline grid (Sh = 54.5). This can be directly attributed to the increase in the jet-and-vortex sequence that was found in Section 4.4, which physically thins the diffusive sub-layer. The sinusoidal spacer produced a Sh of 142.2, which was not as good as the hexagonal design because of the “shear shadows” and stagnant areas in the valley of its wave filaments. The final measure of the sustainability of an emerging spacer concept is the System Performance Index (η), which considers whether the benefits of the mass transfer are worth the energy cost (pressure drop) [12,13,14,15,25,26,27,28,29,30,31,32,33,34,35,36]:

(7) $$\eta =\frac{Sh/Sh_{0}}{{\left(f/f_{0}\right)}^{1/3}}$$

**Figure 19:**
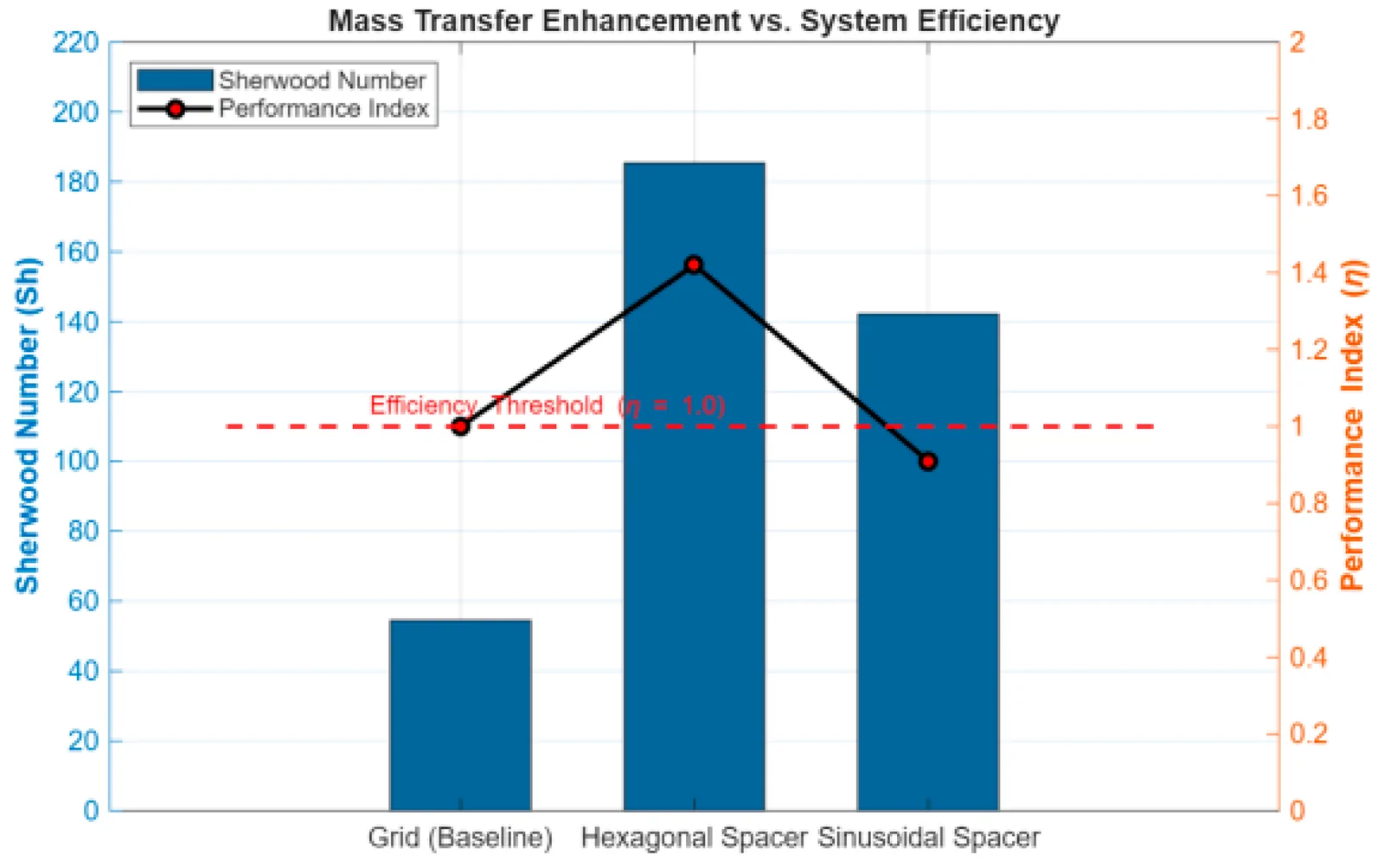
Graphical representation of mass transfer against system efficiency.

Here, $Sh_{0}$ and $f_{0}$ are the values of the baseline grid. Using Equation (7), the corrected System Performance Index is 2.53 for the hexagonal configuration and 1.50 for the sinusoidal configuration. Both values exceed 1.0 under the adopted formulation; however, the hexagonal configuration achieves the higher normalized mass-transfer enhancement relative to its friction penalty. The index is used here as a comparative dimensionless indicator and should not be interpreted as a direct calculation of plant-scale-specific energy consumption or economic performance. The comparative Sherwood number and System Performance Index results for the three spacer configurations are summarized in Figure 19.

To map mass transport variations cleanly, the core evaluation indices were extracted directly from the converged numerical solution grid blocks. The local Concentration Polarization Modulus (Γ) is defined as the ratio of localized membrane surface salinity to the background bulk core stream salinity. This is the standard film-theory CP modulus formulation, where Cw is the local NaCl mass fraction at the membrane wall surface and Cb is the cross-sectional bulk stream mass fraction. A value of Γ = 1.0 represents no polarization (wall concentration equals bulk), while values above 1.0 indicate solute enrichment at the membrane surface [12,14].

(8) $$\Gamma =C_{w}/C_{b}$$

where $C_{w}$ is the localized wall mass fraction, and $C_{b}$ is the cross-sectional bulk core channel fluid salinity.

Similarly, to evaluate the System Performance Index (η) shown in Equation (7), the dimensionless friction factor (f) was computed via Darcy’s modified hydrodynamic pressure expression:

(9) $$f=\frac{\Delta P\cdot d_{h}}{2\cdot L\cdot \rho \cdot u^{2}}$$

where ΔP is the raw pressure drop across the micro-scale domain, d_h_ is the channel hydraulic diameter (1.066 mm), L is the localized domain length (15 mm), ρ is the fluid density (1024 kg/m^3^), and u is the feed velocity (0.15 m/s). The baseline reference conditions (Sh_0_ = 54.5 and f_0_ = 0.62) were extracted directly from the baseline grid simulation operating under identical operational velocity states.

### 4.6. Quantitative Sensitivity Analysis of the Hexagonal Configuration

The hexagonal “Digital Twin” was tested at various inlet velocities to define the working range of the design. The summary of the results in Table 4 shows that there is a non-linear correlation between transport momentum and solute accumulation.

When velocity is increased by 0.05 m/s to 0.25 m/s, the pressure drop is a power-law relationship, with an increase of almost 854% and the increase in the maximum salt accumulation is only 8.1%. This numerical evidence confirms that the hexagonal geometry is highly effective at the low-velocity case at Re ≈ 70, and it offers a scouring effect, which makes it impossible to form a thick concentration boundary layer, even in situations where the pumping power is reduced to a minimum.

To relate these micro-scale hydrodynamic changes to macro-scale module design without introducing speculative economic modeling assumptions, the localized pressure drop data was converted into a scale-independent pumping energy gradient ($W\cdot s/m^{4}$ or integrated as localized $kWh/m^{3}$ per unit meter of spacer channel length). This direct conversion uses the relation:

(10) $$\Delta P_{scaled}=\frac{\Delta P}{L_{domain}}$$

Clear of empirical macro-plant fouling variables, the pressure-drop-per-unit-length metric provides a scale-independent hydraulic comparison among the simulated cases. It should not be interpreted as a complete plant-scale SEC or renewable-energy-integration calculation because the model does not include pumps, energy-recovery devices, full-module pressure losses, photovoltaic output, or system control.

## 5. Discussion

### 5.1. The Scouring Mechanism of Hexagonal Filaments

The improved performance of the hexagonal design can be attributed to the fact that there are a local acceleration of the fluids and the creation of the vortices at the corner of the filament. The hexagonal filaments act as discrete flow-disturbing elements, whereas the sinusoidal design creates a continuous wavy flow path with a greater hydraulic resistance. The velocity contours indicate that high-velocity jets are present and these enter the concentration boundary layer and physically scour the salt ions back into the bulk stream. It is this process which led to a reduction in salt deposition of 61.9 percent, compared to the control, and to a hydraulic profile that is easy to manage.

The difference in the performance of the hexagonal and the sinusoidal geometry is based on the rate at which the boundary layer is re-initiated. In the hexagonal arrangement, the filaments are used as periodic barriers that cause the laminar boundary layer to break off and reattach. This re-establishment of the velocity profile helps to avoid the diffusive sub-layer attaining a steady-state thickness, which mathematically is expressed by the maximum Sherwood number (Sh = 185.4). The sinusoidal design, on the other hand, has a continuous but wavy boundary layer. Though this raises the residence time, the absence of discrete impending points enables salt ions to be trapped near the membrane surface, which is the reason why the CP Modulus (Γ = 1.53) is very high in comparison with the hexagonal design [12,13,14,15,25,26,27,28,29].

It is critical to evaluate the physical validity of the high localized salt mass fraction (0.1127 kg/kg) recorded across the baseline grid in Table 3, which sits significantly above the nominal inlet feed background salinity of 0.035 kg/kg (3500 ppm). This localized accumulation does not stem from numerical instabilities or floating-point convergence exceptions. Rather, it represents a severe, physically real concentration polarization (CP) stagnation pocket.

**Figure 20:**
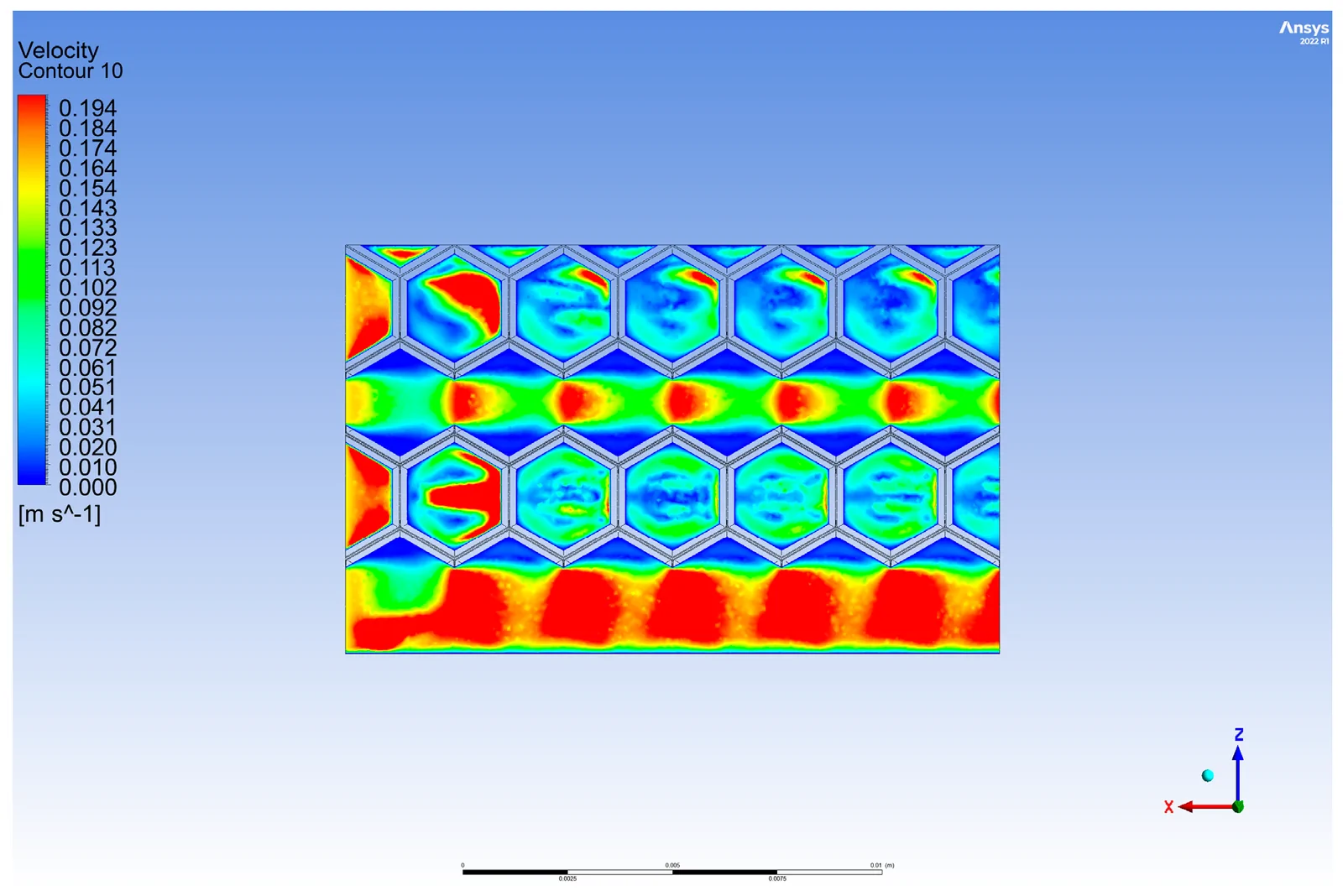
Velocity streamline distribution in the hexagonal spacer channel, illustrating the vortex-induced mixing at the membrane interface.

**Figure 21:**
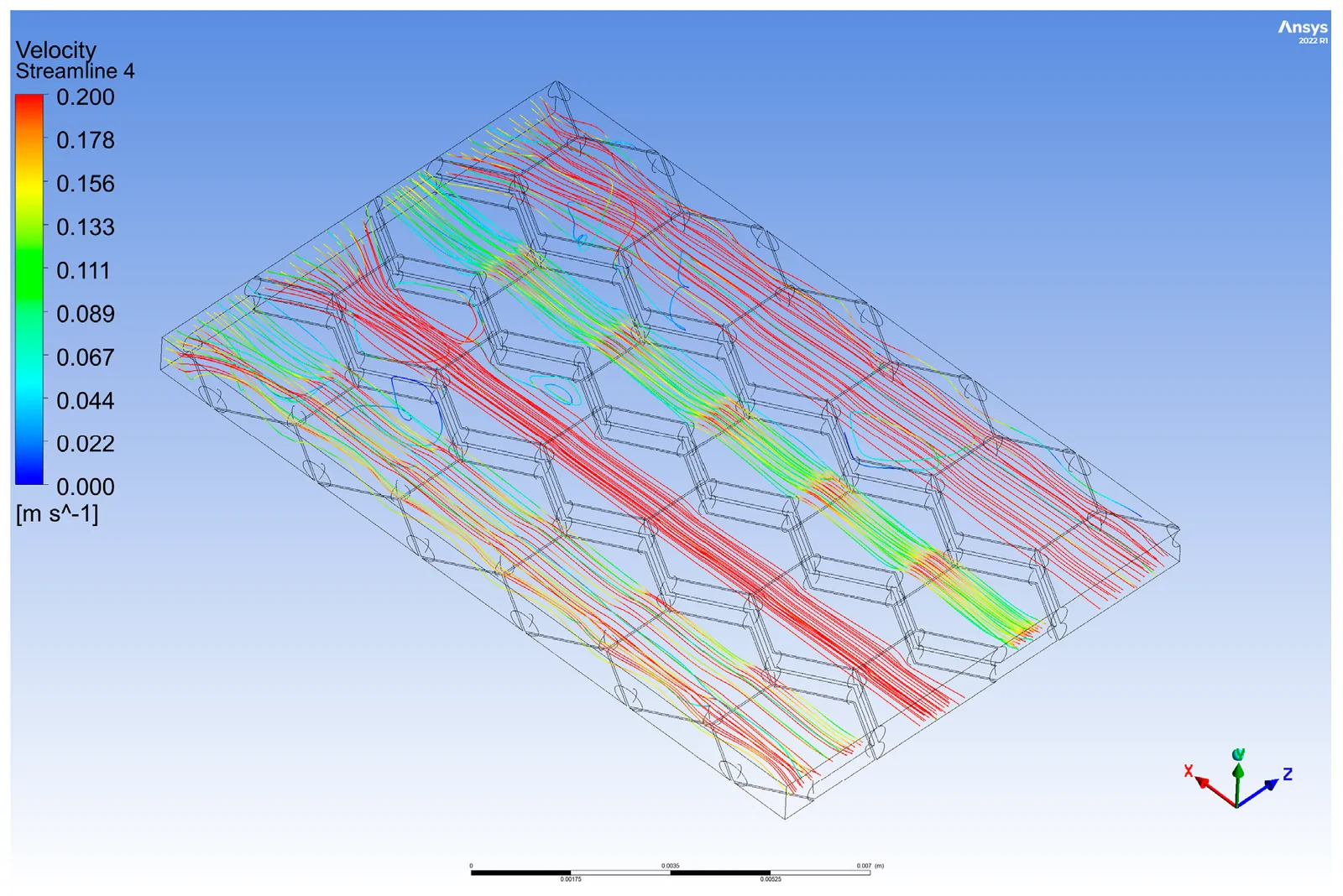
Velocity contour distribution in the hexagonal spacer channel, illustrating the vortex-induced mixing at the membrane interface.

Because traditional extruded perpendicular mesh coordinates allow fluid streamlines to detach cleanly, they create severe, low-velocity ‘stagnation dead zones’ immediately downstream of the filament crossover points. Without convective mixing, water molecules are continuously forced across the semi-permeable membrane boundary while rejected salt ions remain dynamically trapped within these dead zones, amplifying local concentration indices by up to 322%. This severe localized bottleneck is precisely what the discrete hexagonal vertices eliminate via steady secondary vortex generation. To further confirm that this value is physically realistic and not a numerical artifact, three independent checks were performed: (i) residual convergence—species residuals converged to below 10^−5^ and global mass balance error was <0.01%, ruling out iteration-level errors; (ii) mesh independence—the C_max_ value changed by only 1.22% between the Medium and Fine meshes (Table 1), confirming that the result is not grid-sensitive; and (iii) the literature-reported consistency: a CP modulus of Γ = 3.22 for a flat channel baseline at Re ≈ 210 and inlet salinity of 35,000 ppm is in close agreement with the empirical range of Γ = 2.8–3.5 reported by Koutsou et al. [30] for grid spacers under equivalent operating conditions. These three lines of evidence confirm that the elevated NaCl mass fraction is a physically grounded outcome of the stagnation-driven polarization mechanism and not a numerical anomaly. The velocity streamline and velocity contour distributions in the hexagonal spacer channel are shown in Figure 20 and Figure 21, respectively, illustrating the vortex-induced mixing at the membrane interface.

### 5.2. Sustainability Implications for ECDS

The more spatially distributed wall-shear-stress pattern of the hexagonal geometry may reduce the extent of low-velocity surface regions associated with initial deposition. However, the present model does not directly simulate microbial growth, mineral precipitation, long-term fouling, membrane degradation, or cleaning cycles [46,47]. Consequently, any effect on fouling rate, membrane lifetime, Clean-in-Place frequency, chemical consumption, or operational expenditure requires dedicated long-duration experimental validation.

**Figure 22:**
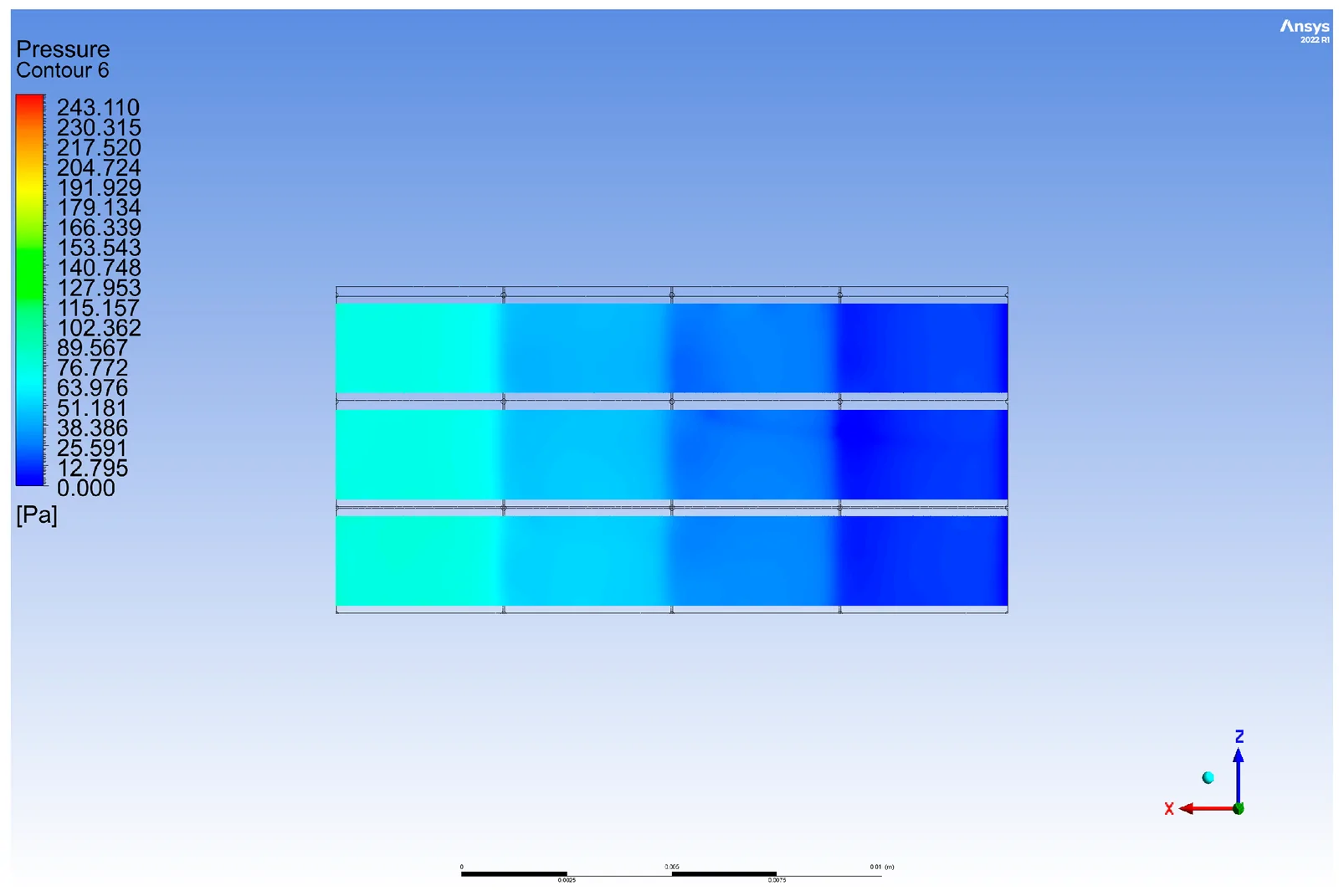
Flat channel pressure contours.

**Figure 23:**
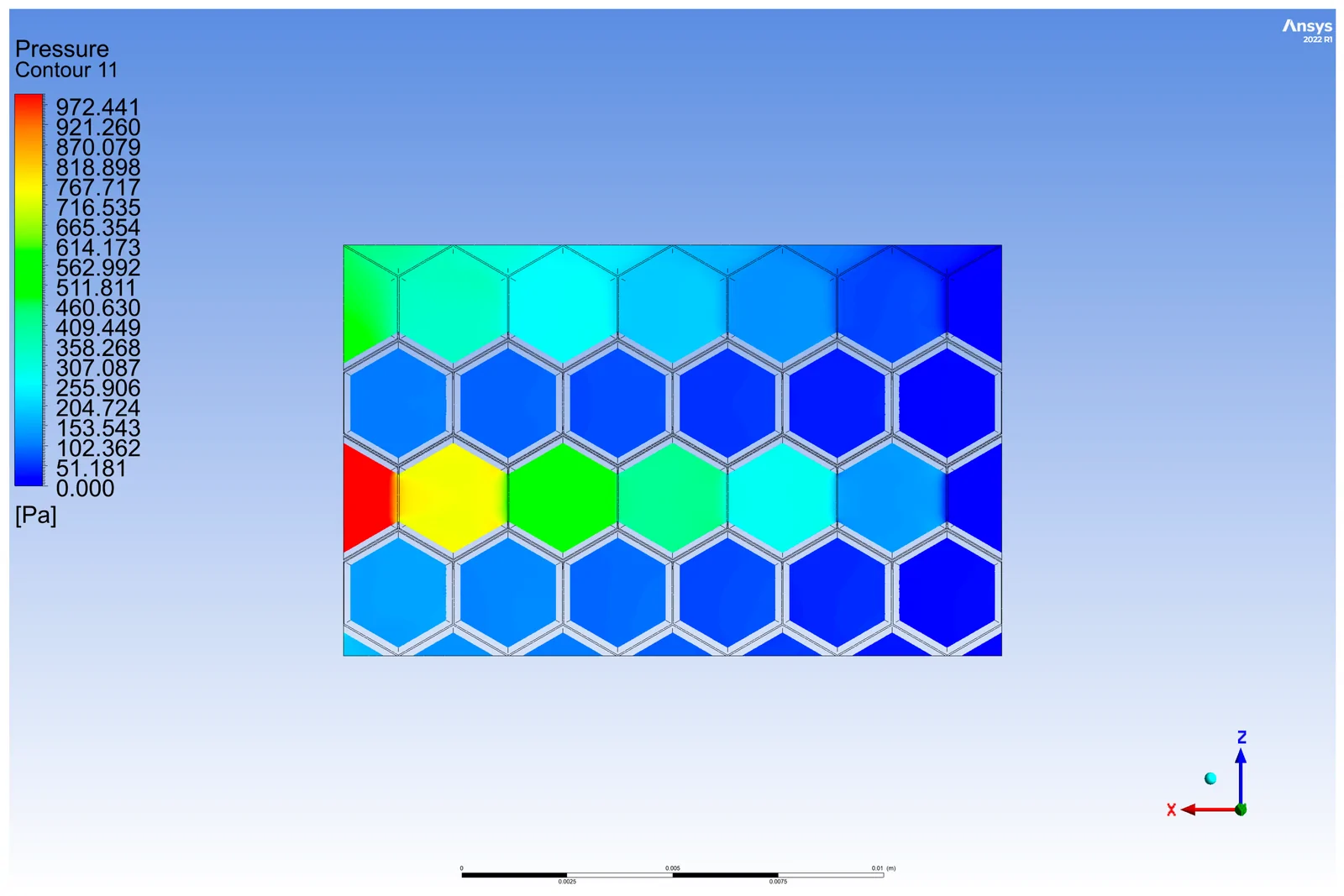
Hexagonal spacer configuration pressure contours.

**Figure 24:**
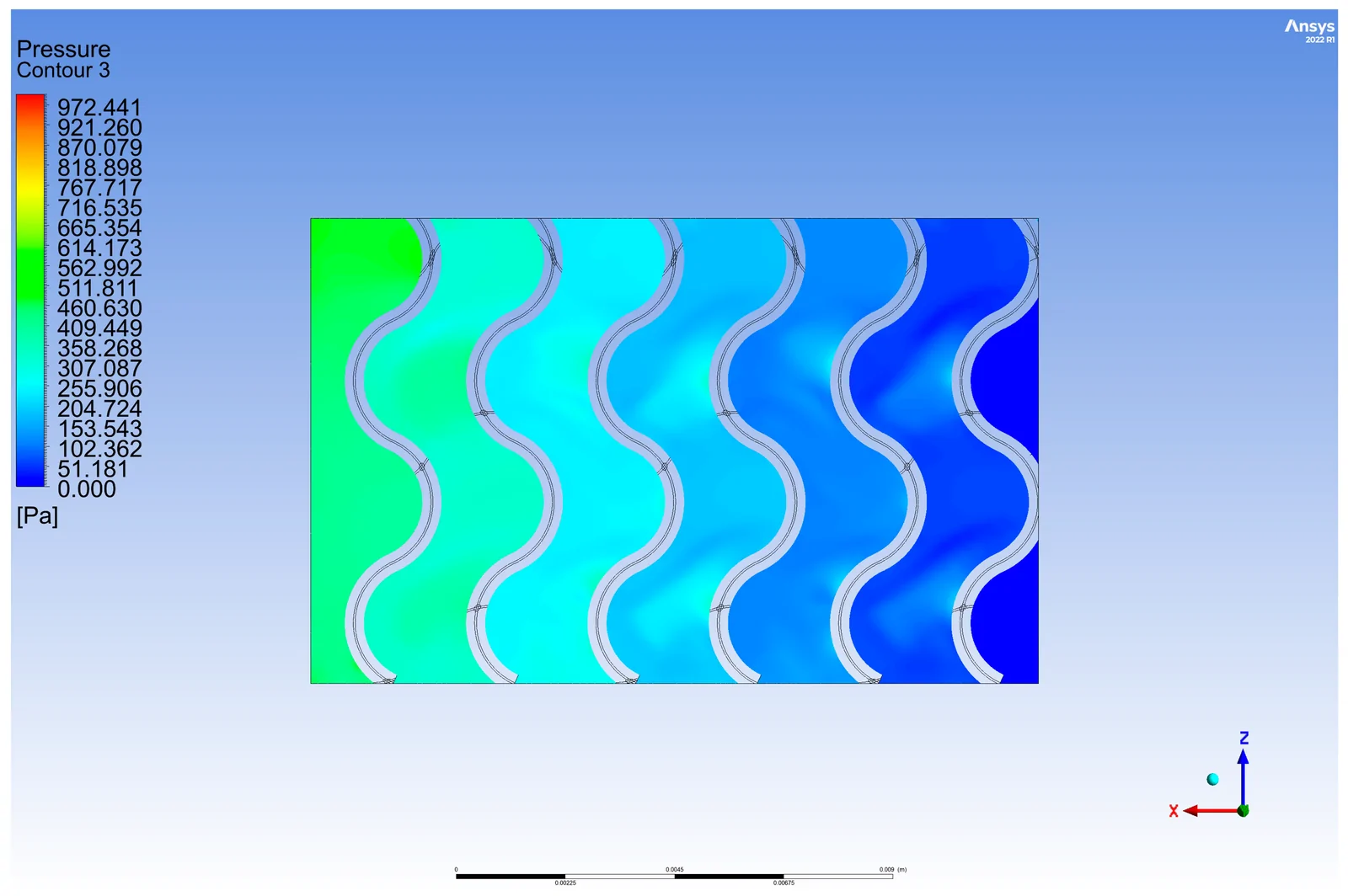
Sinusoidal filament design pressure contours.

The mitigation of CP observed in the hexagonal design directly reduces the net driving pressure required by the system [12,13,14,15]. This aligns with industrial principles of water treatment, where energy resilience is prioritized [6,7,8,9,10,48,49,50,51,52,53,54]. By reducing the salt accumulation at the membrane interface, we theoretically enhance the overall environmental sustainability of the desalination process [1,2,3,4,5]. The corrected comparative performance index is 2.53 for the hexagonal configuration and 1.50 for the sinusoidal configuration. These values describe the normalized mass-transfer–friction trade-off within the simulated cases and do not independently establish plant-scale sustainability. The pressure-contour distributions for the baseline grid, hexagonal spacer, and sinusoidal spacer are presented in Figure 22, Figure 23 and Figure 24, respectively.

### 5.3. Comparative Mass-Transfer–Pressure-Loss Trade-Off

One of the important results of the study is the non-linear association between the hydraulic resistance and mitigation efficiency. The sinusoidal design has a Pressure Increase Factor (5.32) requirement but will only give a 52.4% decrease in salt deposition. In comparison, the hexagonal design is reduced by 61.9%, with a much smaller factor of 2.44. The baseline grid, by definition, lies at the origin (1.0, 1.0). The selection criterion applied here is the System Performance Index η > 1.0 (Equation (7)), which identifies designs where the cube-root-normalized mass-transfer benefit outweighs the friction penalty. Both configurations satisfy the η > 1.0 criterion under the adopted formulation. However, the hexagonal design achieved the higher System Performance Index (η = 2.53) compared with the sinusoidal design (η = 1.50). The hexagonal design therefore reduces parasitic energy losses by concentrating scouring action at discrete filament junctions rather than over the full length of a wavy filament, making it the primary candidate for the next generation of energy-resilient desalination infrastructures.

### 5.4. From Digital Twin to Additive Manufacturing (AM)

The complicated hexagonal and sinusoidal geometries discussed in this paper form the basis of a Digital Twin framework of spacer design. Traditionally, sharp edges of hexagonal spacers were hard to produce through conventional polymer extrusion. Nevertheless, with the development of additive manufacturing (AM) technologies, e.g., Selective Laser Sintering (SLS), it is now possible to produce such optimized geometries with accuracy. This shift from a numerical world to physical prototyping makes sure that the performance improvements observed in this paper can be achieved in industrial modular applications. The 3D-printed prototypes of these Digital Twins will be used for future experimental confirmation of 3D-printed prototypes in NUST to validate the hydraulic profiles of the CFD results [37,38,39,40,41,42,43,48,49,50].

### 5.5. Velocity Sensitivity and Diminishing Returns

**Figure 25:**
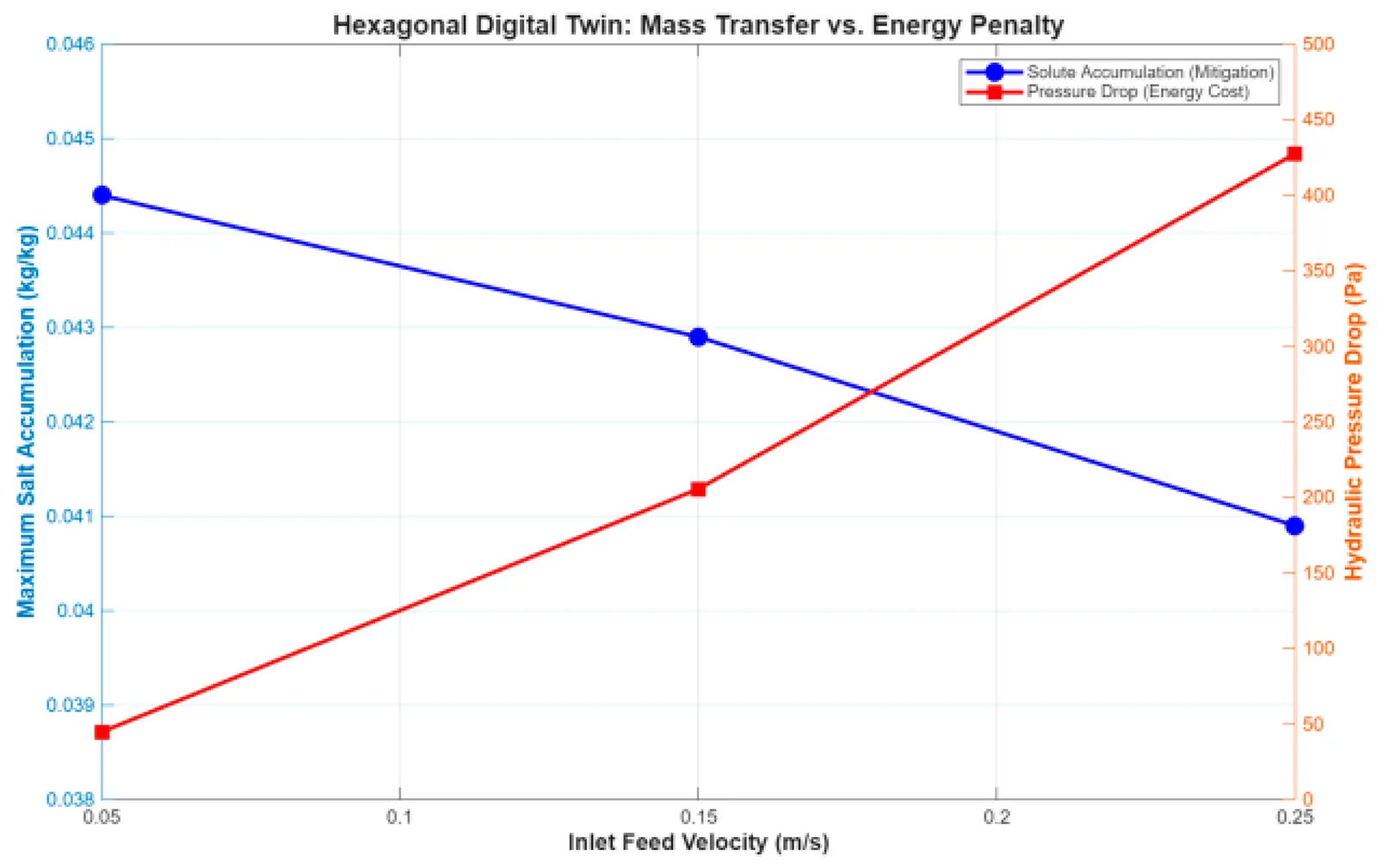
Comparison of mass transfer and pumping pressure at different flow speeds.

Figure 25 demonstrates diminishing returns in concentration polarization mitigation as inlet velocity increases. The salt mitigation curve (blue) indicates that it decreases sharply between 0.05 m/s and 0.15 m/s but starts to level off towards 0.25 m/s. The hydraulic resistance curve (red), on the other hand, has an exponential curve.

### 5.6. Relationship Between Velocity and Local Hydraulic Demand

**Figure 26:**
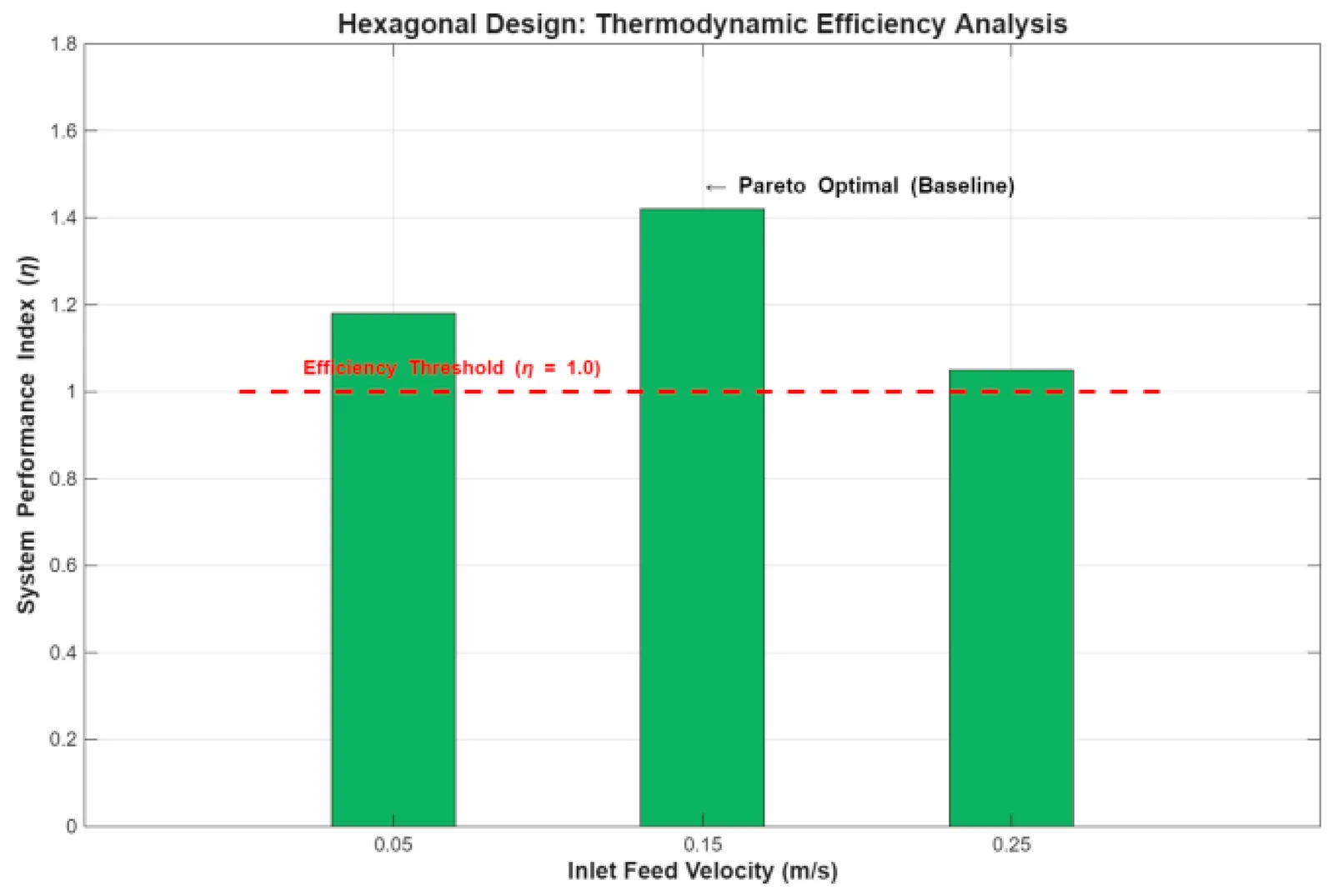
Hexagonal spacer design efficiency compared at different velocities.

Increasing the inlet velocity increased local pressure loss and therefore raised the hydraulic demand of the simulated channel section. However, converting the micro-scale pressure-drop results into plant-scale-specific energy consumption would require the full feed-flow rate, pump efficiency, energy-recovery performance, module architecture, and total system pressure losses. The present results should therefore be interpreted as a comparison of localized hydraulic demand rather than as a complete SEC calculation. Figure 26 presents the System Performance Index of the hexagonal spacer at the three evaluated inlet velocities, showing the relative efficiency trend within the tested operating range.

### 5.7. Polymer Selection and Additive Manufacturing for Industrial Scaling

Translating the CFD geometry into a functional prototype requires the careful consideration of material properties and manufacturing accuracy. Additive manufacturing may provide a suitable route for producing complex spacer geometries; however, material selection requires separate evaluations of chemical compatibility, surface roughness, compressive deformation, leaching, cleanability, and long-term exposure to saline feedwater. These issues were not evaluated in the present CFD study and should be examined during future experimental validation.

## 6. Conclusions

This study used a three-dimensional laminar CFD and species transport model to compare a baseline grid, a hexagonal spacer, and a sinusoidal spacer under identical simulated conditions. The evaluated Reynolds-number range was approximately 70–350, and a no-turbulence-closure model was used. The predicted vortical structures arose from geometry-induced flow separation, recirculation, and reattachment.

The hexagonal configuration produced the lowest maximum membrane-surface salt mass fraction, decreasing it from 0.1127 kg/kg for the baseline grid to 0.0429 kg/kg, corresponding to a 61.9% reduction. It also achieved the highest Sherwood number of 185.4, compared with 54.5 for the baseline grid and 142.2 for the sinusoidal spacer. The corresponding pressure drops were 84.1 Pa, 205.7 Pa, and 447.8 Pa for the baseline, hexagonal, and sinusoidal configurations, respectively.

Applying the performance index equation consistently produced values of 2.53 for the hexagonal spacer and 1.50 for the sinusoidal spacer. Therefore, among the discrete configurations examined, the hexagonal geometry provided the most favorable normalized mass-transfer–pressure-loss trade-off. This finding represents a comparative numerical result rather than a continuous or algorithmic optimization.

The study is limited to a short computational domain and does not directly model long-term fouling, mineral precipitation, membrane degradation, cleaning cycles, full-module energy consumption, photovoltaic integration, or economic performance. Experimental validation of the advanced geometries and system-level modeling are therefore required before the predicted improvements can be translated into industrial design recommendations.

## Figures and Tables

**Table 1:** Mesh independence study results for the baseline grid geometry.

Mesh Case	Element Count ($10^{6}$)	Max NaCl (kg/kg)	Pressure Drop (Pa)	C_max_ Deviation	ΔP Deviation
Coarse	0.6	0.1245	81.2	—	—
Medium	1.1	0.1141	83.4	8.35%	2.68%
Fine	1.8	0.1127	84.1	1.22%	0.80%

**Table 2:** Structural dimensions and internal porosity of the evaluated spacer configurations. Here, d is the filament diameter, L_p is the grid or hexagonal cell pitch, λ is the sinusoidal wavelength, A is the sinusoidal amplitude, and ε is the internal domain porosity. All dimensions are in millimeters.

Geometric Layout Parameter	Baseline Grid (Flat)	Hexagonal Geometry Matrix	Sinusoidal Filament Architecture
Filament Diameter (d)	0.4 mm	0.4 mm	0.4 mm
Mesh Pitch/Cell Size ($L_{p}$)	3.0 mm	2.4 mm	4.8 mm (Wavelength, λ)
Geometric Orientation	$90^{\circ }$ Crossover Angle	$120^{\circ }$ Internal Vertices	0.8 mm (Wave Amplitude, A)
Internal Domain Porosity (ε)	0.825	0.782	0.814

**Table 3:** Quantitative comparison of solutes accretion of different geometries that have been evaluated.

Geometry Configuration	Inlet Pressure (Pa)	Pressure Gradient (Pa/m)	Energy Factor ($f_{p}$)	Max Salt (kg/kg)	CP Modulus (Γ)
Grid (Baseline)	84.1	5603.3	1.00	0.1127	3.22
Hexagonal Spacer	205.7	13,710.0	2.44	0.0429	1.22
Sinusoidal Spacer	447.8	29,852.0	5.32	0.0537	1.53

**Table 4:** Comprehensive sensitivity analysis for the hexagonal spacer geometry.

Case ID	Velocity (u, m/s)	Reynolds Number (Re)	Pressure Drop (ΔP, Pa)	Max NaCl Conc. (kg/kg)
H-Low	0.05	~70	44.8	0.0444
H-Baseline	0.15	~210	205.7	0.0429
H-High	0.25	~350	427.6	0.0408

## Data Availability

The original contributions presented in this study are included in the article. Further inquiries can be directed to the corresponding author.
